# Design Principles of Nanosensors for Multiplex Detection of Contaminants in Food

**DOI:** 10.1002/smll.202412271

**Published:** 2025-05-07

**Authors:** Yang Zhang, Shipeng Gao, Haoran Li, Tianxi Yang, Kaiyi Zheng, Zhing Ming Guo, Jiyong Shi, Xiaowei Huang, Xiaobo Zou, Pierre Picchetti, Frank Biedermann

**Affiliations:** ^1^ School of Food and Biological Engineering Jiangsu University 301 Xuefu Road Zhenjiang 212013 China; ^2^ School of Electrical and Information Engineering Jiangsu University 301 Xuefu Road Zhenjiang 212013 China; ^3^ Food, Nutrition and Health, Faculty of Land and Food Systems The University of British Columbia Vancouver BC V6T 1Z4 Canada; ^4^ Institute of Nanotechnology (INT) Karlsruhe Institute of Technology (KIT) Kaiserstrasse 12 76131 Karlsruhe Germany

**Keywords:** food safety, multiplex, multiplex detection, nanosensors, nanotechnology

## Abstract

The rapid and cost‐effective detection of food contaminants such as toxins and pathogens is a major challenge and a key concern for food safety. To this end, innovative, fast, cost‐effective, and easy‐to‐use sensors must be developed at the point where food is produced, distributed, and consumed. Therefore, timely detection and response to food contaminants can improve human health and reduce economic burden. However, affordable sensor technologies with specificity, sensitivity, and speed are required, which can be used by non‐specialized personnel and enable high throughput analysis. In this respect, advances in the development of nanoparticle‐based sensors, i.e., nanosensors, have shown the potential to provide the much‐anticipated versatile sensors. In addition, multiplex detection, i.e., the ability to detect multiple targets simultaneously, is another strategy facilitated by nanoparticle‐based sensors and will enable further improvements in sensor performance that are important for developing effective monitoring. This review summarizes the nanosensors for multiplex sensing of food samples with respect to hazardous contaminates reported over the past few years. In addition, special attention is paid to providing the reader with promising design principles and the current performance of the sensitivity and selectivity of such sensors for practical requirements, thereby inspiring new ideas for developing further advanced systems.

## Graphical abstract

This review discusses recent advancements in nanoparticle‐based nanosensors for multiplex detection of food contaminants, focusing on toxins and pathogens. It highlights the design principles, sensitivity, and selectivity of these sensors, offering insights into their practical applications for food safety monitoring and inspiring future innovations.

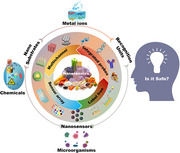

## 1 Introduction

**General remarks**. Assuring the safety and quality of processed and primary food ingredients is an ongoing global challenge. The World Health Organization (WHO) report indicates that 600 million cases of foodborne illness are reported each year.^[^ ^1^, ^2^ ^]^ The number of incidents is expected to increase due to the anticipated increase in global food demand, which results from the expected increase in the global population combined with socioeconomic changes, such as the improvement in living standards in many parts of the world.^[^ ^3^ ^]^ To ensure food safety and reduce economic damage caused by contaminated food, the development of rapid, affordable, and easy‐to‐use sensors is crucial to enable cost‐effective detection of contaminations at all stages, from production to consumption. This demand requires rapid testing for food contaminants, which can pose an overwhelming task for regulatory laboratories that rely on time‐consuming and resource‐intensive chemical and biological testing methods, such as high‐performance liquid chromatography (HPLC), mass spectrometry (MS) and antibody or enzyme‐based sensors (e.g., ELISA tests).^[^ ^4^, ^5^ ^]^ Globalization, economic rivalry, and security threats exacerbate these challenges and make the food supply chain increasingly vulnerable to contamination by harmful chemical and biological substances.

The past food safety crises have enhanced standards of food safety worldwide, but it would be unrealistic to assume that future food safety crises won't occur. For example, the melamine contamination in pet food and infant formula, which caused severe health issues in animals and young children, was difficult to foresee.^[^ ^6^ ^]^ Melamine and cyanuric acid were illegally added to these products to falsely boost protein content because regulations at the time relied on hydrolyzing food with acid and measuring NH_3_, which was assumed to correlate with protein levels.^[^ ^7^ ^]^ The problem was uncovered when chromatography‐MS analysis was used to specifically detect melamine. This crisis highlights the need for more affordable detection methods that target specific molecules and provide more comprehensive information, rather than only relying on HPLC/MS.

Nanosensors are expected to provide new sensor technologies that fulfill some of the required performance characteristics for fast, cost‐effective, and robust sensors.^[^ ^8^, ^9^, ^10^ ^]^ By definition, nanosensors are sensing devices containing nanoparticles, typically 1–100 nm in size, which are critical to the chemical aspect of the sensor design and comprise either the receptor or the transducer element.^[^ ^11^, ^12^ ^]^ In addition, the introduction of new nanosensor technologies in the food sector is sometimes hampered by regulatory, political, legal, economic, environmental, health and safety, and ethical challenges, which have more recently been excellently reviewed and analyzed by Yang and Duncan^[^ ^13^ ^]^ which will not be subject of this review. Nanosensors can detect a wide range of contaminants, including inorganic salts, small organic molecules, proteins, viruses, and pathogens. These sensors can identify chemical substances in all states of aggregation, such as liquid or solid – with ultra‐low detection limits. They can be used in various environments along the food supply chain, from production and processing to retail and consumption, and for a wide range of foods.^[^ ^14^, ^15^, ^16^, ^17^, ^18^ ^]^ The sustained demand for faster, more accurate, and sensitive analytical methods has led to miniaturized and multiplex assays. In the application of nanosensors, multiplexing aims to detect multiple targets simultaneously, which has a significant effect of decreasing time and cost and getting more information from a single sample.^[^ ^19^, ^20^, ^21^ ^]^

In this Review, we will summarize the development of nanosensors with the ability to perform multiplex analysis, which have been applied in foods, focusing on the strategies and principles in multiplex detection. The examples presented herein cover reports that have been published since 2019. We first introduce the general concept of nanosensors. Then we cover the distinctive strategies in multiplex analysis. In this part, we will also assess the recent advances toward making the practical detection of food contaminations possible, including nanosensor‐assays empowered by machine learning (ML). We have divided the analytes into metals, chemicals, and microorganisms and explored the smart nanosensor designs in the following sections. Although some of the analysts are not strictly related to foods, similar design strategies can be readily applied for food analysis. Finally, the perspective of challenges and opportunities in this area is provided as our conclusion.

## 2 The Overview of Nanosensors

### 2.1 The Composition of a Nanosensor

**Figure 1:**
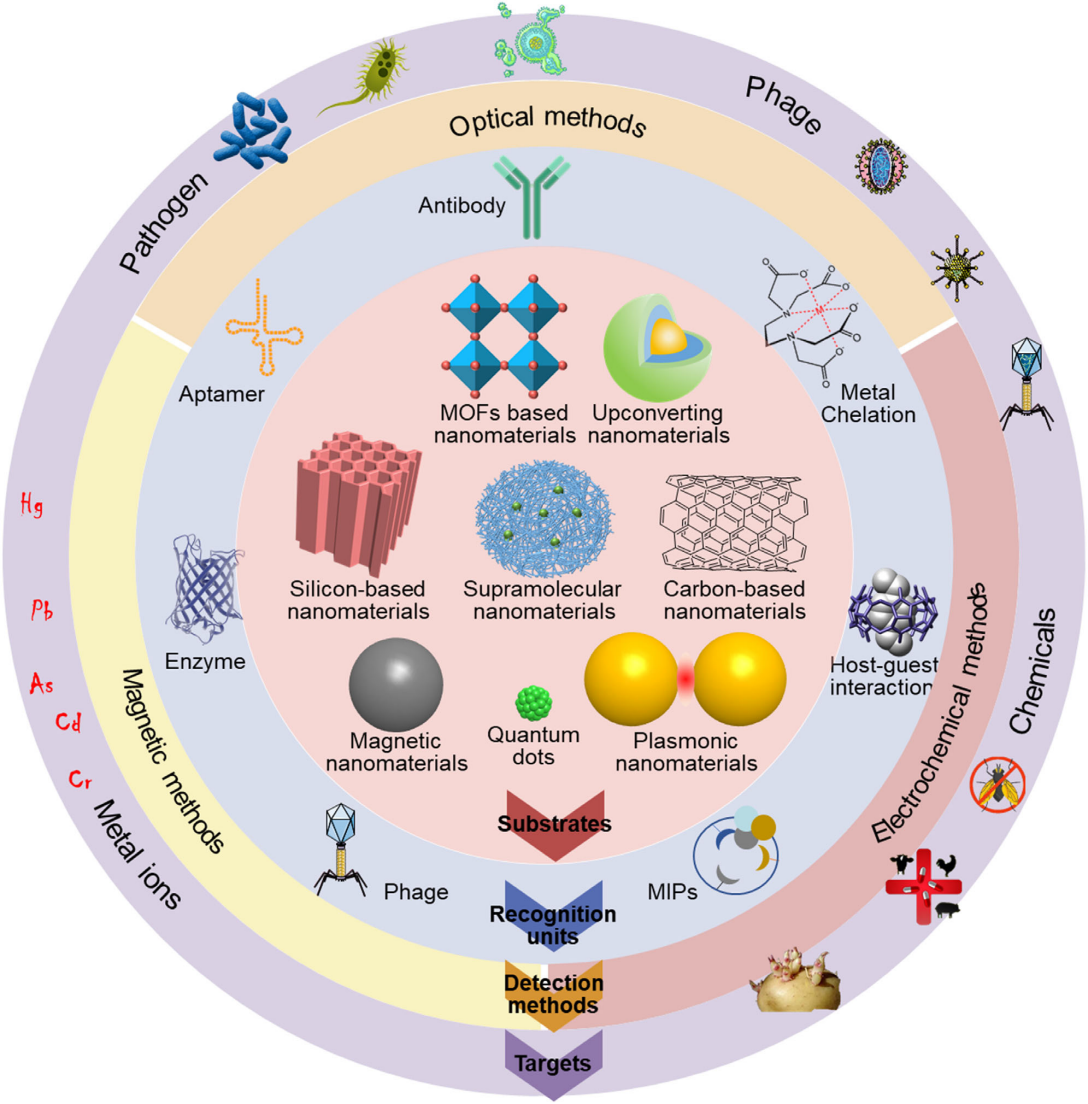
The general compositions of nanosensors, including nano substrates, binding elements, and the detection mechanisms (producing detectable signals) for contaminants in food.

In essence, a nanosensor can be divided into three main elements: a binding site for the analyte (binding element), a signal transducer that converts the binding event of the analyte into a recordable signal, and elements for signal amplification/processing (**Figure** **1**). Often, nanosensors rely on nanomaterials that are conjugated to a binding element (in some publications, this is also called a ‘targeting ligand’ or “receptor”), where the element binds specifically to the target molecule, producing the nanosensor specificity, and the nanoparticles serve as the transducer (generator or detector) of a signal, giving sensitivity. Nanoparticles provided incomparable properties for detection, such as a significant surface‐to‐volume ratio, high reactivity, unique magnetic and optical properties, and enhanced electrical conductivity.^[^ ^22^, ^23^, ^24^, ^25^ ^]^ With the developments of nanotechnology, nanoscale dimensions of nanomaterials, which are magnetic nanoparticles, metal–organic frameworks (MOFs) based nanomaterials, silicon‐based nanomaterials, carbon‐based nanomaterials, plasmonic nanomaterials, etc., enlarge sensitivity by integrating novel transduction principles such as local surface plasmon resonance, superparamagnetic properties, enhanced electrochemical activity into nanosensors.^[^ ^26^, ^27^, ^28^, ^29^, ^30^ ^]^ Noteworthy progress has been made in the development and utilization of nanosensors in food safety, security, medical diagnosis, and environmental protection. Based on the signal transduction mode, nanosensors can be classified into optical nanosensors,^[^ ^31^, ^32^ ^]^ electrochemical nanosensors,^[^ ^33^, ^34^, ^35^ ^]^ and magnetic nanosensors.^[^ ^36^, ^37^ ^]^ The classification into chemical sensors^[^ ^38^, ^39^ ^]^ and biosensors^[^ ^40^, ^41^ ^]^ is also common.

### 2.2 The Mechanism of Producing Detectable Signals

Signal transduction occurs after the binding event of the analyte to a binding site (receptor). There are three commonly used signal‐transduction mechanisms: optical methods, electrochemical methods, and magnetic methods. First, Signal readout based on luminescence uses fluorescent, phosphorescent or chemiluminescent reporter molecules that either emit light that is directly recorded by a detector or that can interact with the nanoparticle on which they are located prior to signal readout through energy transfer processes to further modulate the light emission, e.g. by changing the wavelength (color) or intensity of the emission.^[^ ^42^, ^43^ ^]^ Luminescent reporter molecules can be fluorescent dyes^[^ ^43^ ^]^ or polymers,^[^ ^44^ ^]^ lanthanide complexes,^[^ ^45^ ^]^ and luminol‐H_2_O_2_ system,^[^ ^46^ ^]^ to the nanoparticles themselves after combination with analytes, such as quantum dots,^[^ ^47^, ^48^ ^]^ MOFs^[^ ^49^, ^50^ ^]^ and upconverting nanoparticles.^[^ ^51^, ^52^, ^53^, ^54^, ^55^ ^]^ A wide range of fluorescence signals depend on various mechanisms, including Förster resonance energy transfer (FRET), electron or charge transfer,^[^ ^56^, ^57^ ^]^ photoinduced electron transfer, monomer excimer formation, and the rigidity effect.^[^ ^58^ ^]^ Spectroscopic detection methods that make use of plasmonic effects, such as surface plasmon resonance spectroscopy (SPR) and surface‐enhanced Raman scattering (SERS), also play an important role. These methods detect signals caused by changes in the dielectric constant of the medium surrounding the sensor. Furthermore, Raman signals can be enhanced by local surface plasmon resonance.^[^ ^59^, ^60^, ^61^, ^62^, ^63^ ^]^ Second, electrochemical techniques such as voltammetry or amperometry alongside other methods such as charge potential or accumulation/potentiometry, conductivity/conductometry, and resistance and reactance/impedance spectroscopy, make them a popular choice for signal transduction.^[^ ^64^, ^65^, ^66^ ^]^ Third, the transverse relaxation time (*T_2_*) time of water around magnetic nanoparticles will be tuned by the tarte‐induced variation of nanoparticles (aggregation or disaggregation). Based on this, the magnetic relaxation switching assay(MRSw) has been used to detect pathogens, organic pollutants, metal ions, etc.^[^ ^36^, ^67^, ^68^, ^69^ ^]^ This is similar to the colorimetric assay, which is based on the assembly of plasmonic nanoparticles.^[^ ^70^, ^71^ ^]^ But, compared to the optical methods, MRSw is unimpressive. In this method, there is only one kind of signal, the intensity of T_2_, so it is important to utilize magnetic nanomaterials with high magnetism.^[^ ^72^ ^]^ In addition, molecular recognition elements are often attached to nanoparticles, which improves immobilization strategies in the development of composite materials. These strategies are critical for device fabrication, as they increase surface area and enable complex signal transduction cascades from the probe to the nanoparticles. Common methods for this binding include covalent bonding, biotin‐streptavidin interactions, and physical adsorption.^[^ ^73^, ^74^ ^]^

### 2.3 The General Methods of Conjugating a Binding Element with a Nano‐Substrate

Nanoparticles require modifications that provide analyte‐specific applications. Functionalization techniques provide uniform and tunable chemistry to nanomaterials, for example, ligand exchange, which is used to transfer hydrophobic surfactants from magnetic nanoparticles to hydrophilic ligands through dative chemistry.^[^ ^75^ ^]^ Additionally, the orientation of molecules on nanoparticles influences sensor specificity and sensitivity.^[^ ^76^, ^77^ ^]^ In this part, we will focus on the conjugation of recognition elements including organic molecules and biomolecules (**Table** **1**). These methods can be divided into covalent, coordinative, and noncovalent functionalization, which are key strategies for creating chemically stable conjugations in the functionalization of nanoparticles. Condensation reaction is a popular method for surface functionalization. One of the oldest methods is the condensation of amines and aldehyde between proteins and glutaraldehyde.^[^ ^78^ ^]^ While, now, researchers use amines, carboxylates, and thiols as functional groups frequently.^[^ ^79^, ^80^, ^81^ ^]^ Meanwhile, the common cross‐linkers are succinimidyl 4‐(N‐maleimidomethyl)cyclohexane‐1‐carboxylate (SMCC) (thiols/amines) and 1‐ethyl‐3‐(3‐(dimethylamino)propyl)carbodiimide/N‐hydroxysuccinimide (EDC/NHS) (carboxylates and amines).^[^ ^82^, ^83^ ^]^ Click chemistry approaches such as the copper‐catalyzed azide‐alkyne click reaction (CuAAC),^[^ ^84^, ^85^ ^]^ the strain‐promoted azide‐alkyne click reaction (SPAAC)^[^ ^86^ ^]^ display the superiorities of high yield under simple reaction conditions, inoffensive/minimal byproducts, simple product isolation, etc., also being found widespread applications in the functionalization of nanosensor.^[^ ^87^, ^88^ ^]^ Sometimes, the reactions between nucleophiles and isocyanates were used.^[^ ^89^ ^]^ These two methods prefer to indirect conjugation of recognition element of the nanoparticles surface, while the dative bonds is a direct connection to the surface. A well‐known example is Au‐thiol chemisorption, e.g., the aptamers in SERS sensor.^[^ ^90^, ^91^ ^]^ Noncovalent attachments utilize relatively weak coordination bonding, and stability is characterized by equilibrium dissociation constants. Bioconjugation methods based on avidin‐biotin complex interactions are also widely used as the strong affinity with an association constant (K_a_) of 10^15^ M^−1^.^[^ ^92^ ^]^ These methods conjugate molecules effectively and only minimally affect their properties.^[^ ^93^, ^94^ ^]^ Other noncovalent interactions comprise of host–guest interactions or hydrophobic–hydrophilic interactions.^[^ ^95^, ^96^, ^97^ ^]^ While the aforementioned strategies are based on chemical reactivity and well‐defined supramolecular interactions, nanoparticles can also be functionalized by less specific physical adsorption processes. These noncovalent processes include van der Waals forces, hydrophobic interactions, electrostatic interactions, and hydrogen bonding.^[^ ^98^, ^99^ ^]^ Electrostatic attachment is the simplest nanoparticle modification approach, which is driven by the oppositely charged species.^[^ ^100^ ^]^ Although these methods are relatively simple and effective, they lack the specificity and orientation control of other methods and are generally less stable as they are sensitive to environmental factors such as pH, temperature, and salt concentration.

**Table 1:** Examples of commonly used surface conjugation methods.

Type	Surface functionalization used	Recognition element	Metal surface materials	Sensing application	Sensing methodology	Performance	Refs.
Covalent interaction	Carboxy‐amine reaction via EDC/NHS	Aptamer	Carboxy‐modified polystyrene microspheres	S1‐protein, SARS‐CoC‐2, gp120, HIV	UV/vis spectroscopy	12.2, 8.0 ng/mL, 2 cells/mL, 6 cells/mL	[101]
	thiol‐amine reaction SMCC	LPS binding peptide	(GT)_20_‐NH_2_/SWCNT	P. aeruginosa and S. aureus	Fluorescent nanosensors array	Differentiation of two pathogens	[102]
	“click” chemistry	DNAzymes	Capture DNA with carbon triple bond	Mg^2+^ and Pb^2+^	Electrochemical method	34 pM, 166 pM	[103]
Coordinative interaction	Dative bonding (Ag‐S)	Small molecule with cross reactivity	Silver nanoparticles	Wine flavors: 3‐mercapto hexyl acetate, limonene, linalool, menthol, 3‐mercapto‐1‐hexanol	SERS sensor array	Multiplex profiling of five flavors with 100% accuracy at ppm level	[104]
Noncovalent interaction	Biotin‐avidin interaction	Antibody and aptamer	Gold nanoparticles	Biomarker targets (miRNA‐1 and prostate‐specific antigen)	Imaging	50 fM, 1 pg/µL	[105]
	Host–guest	cucurbit[n]urils (CBn, n = 7, 8)	Gold nanoparticles	methylxanthine drug isomers	SERS label free method	50 nm	[106]
	Electrostatic interaction and π‐π stacking	antibody	2D MXene‐Ti_3_C_2_T*_x_* nanosheets	Cancer biomarkers	Electrochemical methods	1 ng/mL	[107]

### 2.4 The Outline of Nanosensors with the Ability of Multiplex Detection

The ASSURED criteria,^[^ ^108^ ^]^ published by the WHO, include characteristics that are considered ideal for point‐of‐care sensors: Affordable, sensitive, specific, user‐friendly, fast and robust, device‐free, and available to the end user. These benchmarks are widely regarded as essential goals for the design and development of point‐of‐care sensors.^[^ ^109^ ^]^ In food safety, the idea of point‐of‐contamination detection has also been incorporated, adopting the hazard analysis‐critical control point (HACCP) strategies.^[^ ^108^, ^110^ ^]^ However, given the unforeseen threats and long supply chains in food safety, detection methods should be inexpensive and easy to implement, but also have the capability for multiplex detection. An often‐encountered major disadvantage of many nanosensors currently under investigation, which are designed for the detection of an analyte, is their low efficiency and capability of anti‐interference in detecting contaminants in real samples. This is because interfering factors (competing analytes, matrix components that affect the nanosensor) in the sample can heavily affect selective detection.^[^ ^111^, ^112^, ^113^ ^]^

The ability to quantitatively detect multiple contaminants such as metals, chemicals, and microorganisms is therefore a promising approach to solving the problem of selectivity in analytical chemistry. This approach requires first the development of high‐throughput nanosensors and secondly the implementation of sophisticated data analysis tools. These tools are essential to obtain distinct fingerprint detection patterns for numerous analytes with multiple signal readouts. Compared to analyzing a single analyte, multiplex detection enables the identification of multiple target analytes simultaneously in complex sample matrices, saving samples, and reducing the number of analytical techniques used, saving overall time, costs, and sample volumes. For example, the multiplex polymerase chain reaction (PCR) assay provided easier operation and shorter assay time (only 3h) in the detection of four different pathogenic retroviruses, whereas traditional methods needed 3 days.^[^ ^114^, ^115^, ^116^ ^]^ Recently, Zhang's group reported that a paper chromogenic array assisted by machine learning could detect viable *Escherichia coli* and other viable pathogens simultaneously, without sample preparation steps such as enrichment, culturing, and incubation procedures.^[^ ^117^ ^]^ Generally, binding events of target molecules can be distinguished by receptor‐functionalized nanoparticles, while high‐density analysis with a variety of targets can be achieved by increasing the number of sensor units. In this strategy, optical encoding is the most popular as it has flexible and convenient properties. For example, by doping multicolor quantum dots with different levels into mesoporous silica, more than 30 types of output signals were distinct with two colors, while 1000 signals could be obtained with 3 colors.^[^ ^118^ ^]^ These methods could produce barcode particles with high flexibility to detect multiple analytes in a microarray, for example, upconversion nanoparticles doped with different rare earth ions and nanocomposites with various quantum dots.^[^ ^119^, ^120^, ^121^ ^]^ The nanosensors based on these nanobarcodes can be supposed to be the development of ‘one‐to‐one’ detection, combining several sensor units in an array that target special analytes to realize multiplex detection.^[^ ^122^ ^]^ With the same principle, assembling several individual electrodes within an array enables multiplex electrochemical detection of multiple targets, so an electrochemical sensor can also be used for multiplex sensing with the strategy of the sensor array.^[^ ^123^, ^124^, ^125^, ^126^ ^]^ However, in some applications especially fast detection, for example, the primarily on‐site test with a large number of samples, a qualitative but rapid Yes/No result is more necessary. This imposes an undue burden on quantitative conclusions according to the sensing results of multiple targets.^[^ ^127^, ^128^ ^]^ This kind of analysis can be carried out with Boolean logic, where ‘1’ and ‘0’ are used to express with and without the target.^[^ ^129^ ^]^ Accordingly, depending on whether the nanosensor unit could detect the analyte or not, that is, the output signal with on or off status, the logic gates strategy was used to encode the output signals when multiple analytes were input.^[^ ^130^, ^131^ ^]^ Generally, the nanosensor array with barcode particles can quantitatively perform the multiplex assay, while the nanosensor with logic gates can only qualitatively perform multiplex sensing qualitatively, at least to a certain degree.^[^ ^132^, ^133^, ^134^, ^135^ ^]^ A further example, the ultimate signal could also be encoded by the signals from each sensing channel with interactive reaction.^[^ ^136^ ^]^ Under this circumstance, the intensity of signals would change under various analytes, and the composite signal would be identified by a ML algorithm.^[^ ^137^, ^138^, ^139^, ^140^ ^]^ Most of these methods need recognition elements in the fabrication of nanosensors with covalent or noncovalent interactions, and the signals are from molecule tags. Fortunately, SERS provides ‘fingerprint’ information of the target, which contains fundamental chemical information, also providing the possibility of multiple discrimination.^[^ ^141^, ^142^, ^143^, ^144^ ^]^

A typical data analysis method for quantitative detection is based on standard curve methods, especially linear regression and polynomial regression methods are used for quantitative detection mostly.^[^ ^145^, ^146^, ^147^, ^148^, ^149^ ^]^ Mostly, the signal discrimination is based on the variation in color, spectral intensity, spectral position, fluorescence lifetime, etc. However, traditional data analysis methods have poor performance with convoluted signals resulting from food matrices that contain many interferences and multiple targets. Along with further research, ML algorithms are used to perform in‐depth feature analysis among complex signal variances and construct accurate prediction models.^[^ ^150^, ^151^, ^152^ ^]^ The application of ML algorithms facilitates pattern recognition, higher throughput, and data processing of convoluted signals. They are thought to be a powerful tool for enabling practical applications of nanosensors. Up to now, in environmental, chemical, and biomedical sensing, ML algorithms have been extensively used.^[^ ^153^, ^154^, ^155^ ^]^

ML algorithms can accelerate multiplex correlative data processing and pattern recognition of intricate output signals.^[^ ^156^, ^157^, ^158^ ^]^ Moreover, ML algorithms enhance nanosensor detection accuracies by reinforcing predictive analytics.^[^ ^104^, ^159^, ^160^ ^]^ Under these circumstances that signal variances resulting from concurrent changes, ML algorithms are more advanced than standard cure methods, with the functions of data processing, classification, and prediction.^[^ ^161^, ^162^ ^]^ In detail, ML algorithms can elucidate complex signals by reducing data dimensionality and assessing the feature importance of high dimensional data, for example, principal component analysis (PCA) and *F*‐scores.^[^ ^163^, ^164^ ^]^ Further, the most primitive motivation for utilizing ML algorithms is the capability of rapid predictions for future data, which is the same as the application of linear regression. Here the ML algorithms used are neural networks, support vector machines (SVM), etc.^[^ ^117^, ^165^ ^]^ The codes of ML algorithms are complex for researchers who have the background of chemistry and biology, but fortunately, most of the ML algorithms can be downloaded from GitHub, and can be run by Python, MATLAB, etc. With the increasing popularity of ML algorithms in sensing and multiplex analysis, we will focus on their applications here.

## 3 Design Strategies of Nanosensors for Multiplex Analysis

Conventional detection methods are designed and optimized for a given analyte based on the ‘lock and key’ principle, first described by Emil Fischer.^[^ ^166^ ^]^ Specific molecular recognition elements, in particular antibodies, enzymes, and aptamers, have been developed as receptors for the recognition of individual analytes according to this long‐standing principle. Although great improvements have been achieved, the detection of a single analyte by interaction with the corresponding receptor has some disadvantages that continue to exist. First, it is a great challenge to design receptors for similar analytes with high selectivity. For example, molecular probes that form covalent bonds with their analytes do not exhibit significant differences in reactivity when they encounter interfering substances, leading to false positive.^[^ ^167^, ^168^ ^]^ Synthetic receptors,^[^ ^38^, ^169^ ^]^ which function via noncovalent interactions, often do not provide sufficiently high binding affinities between the analyte and interferents to achieve effective detection for a single analyte. Although aptamers can be developed with theoretically acceptable selectivity, their production, e.g., by SELEX (systematic evolution of ligands by exponential enrichment) methods, remains difficult.^[^ ^170^ ^]^ In addition, synthetic nucleic acid‐based receptors are too expensive and labor‐intensive to produce and suffer from significant stability problems associated with biological media.^[^ ^171^ ^]^ The selectivity problem is not limited to the detection of small molecular contaminants but also extends to the detection of proteins, cells, and bacteria. For example, in the detection of *E. coli* O157:H7, which is a typical foodborne pathogen, the unwanted signals from other bacteria are unignorable, especially those from other *E. coli* serotypes.^[^ ^167^, ^168^, ^172^ ^]^

There is a broad consensus that the use of lock‐and‐key principles for the selective detection of a single analyte is impractical in real‐life scenarios for the identification of contaminants in food, and at the moment only the lateral flow pregnancy tests and the electrochemical glucose biosensor enjoy global commercial success. Therefore, numerous multiplex sensor methods have been developed.^[^ ^173^ ^]^ These can include techniques such as the use of barcode particles and the use of logic gate strategies, analyzing the response of many individual receptors to their corresponding analytes.^[^ ^174^, ^175^ ^]^ Another form of multiplex detection mimics the olfactory/gustatory system.^[^ ^176^, ^177^ ^]^ Here, the analyte interacts with each sensing unit through noncovalent interactions, creating a distinctive “fingerprint” signal based on the sensor array's collective response. This needs to balance the selectivity and affinity of the recognition element with the analyte. Here, due to the high volume and similarity of the signal of each analyte, ML algorithms are widely used to discriminate the signals, for example, PCA was used for pattern recognition.^[^ ^178^ ^]^ Furthermore, the analysis of real‐world samples and the inherent variability among these samples serve to amplify the complexity of the data. Traditional statistical data analyses are inefficient or even obsolete for analyzing data sets from such datasets. This motivates the use of ML for the robust examination of the complex dataset. Here, we will describe each of the strategies and explain how the ML facilitates the data analyses from the aspects of choosing recognition elements and signal transduction combinations.

### 3.1 The Types of Binding Elements

**Figure 2:**
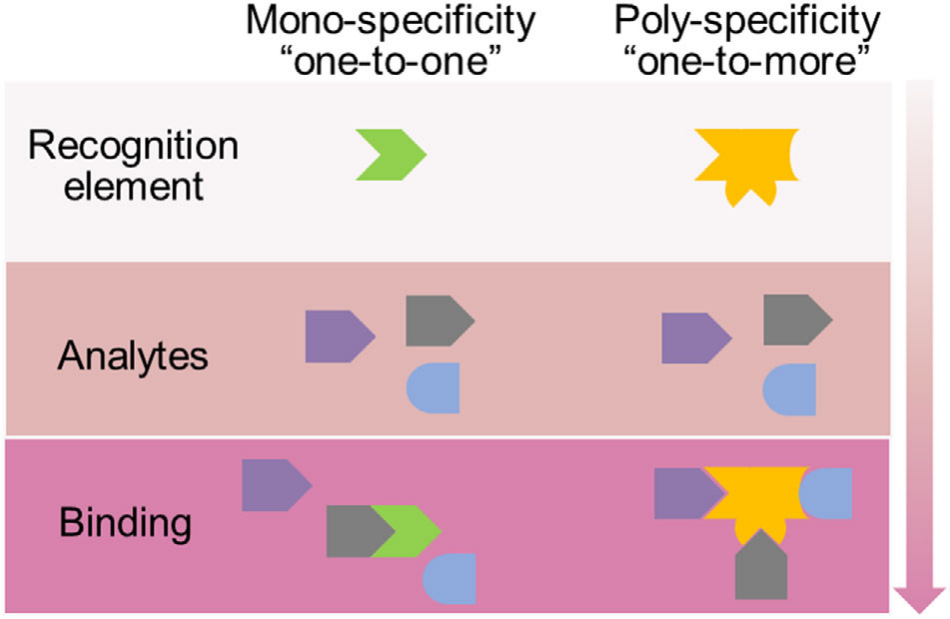
Two types of recognition elements with different specificity.

Binding elements, as the name suggests, are designed to capture the desired target analyte or a group of analytes from a sample. These binding sites can consist of reactive probes that irreversibly bind the analyte by forming strong covalent or coordinative bonds.^[^ ^179^, ^180^ ^]^ For example, the oligopyrroles as ion probes,^[^ ^181^ ^]^ sensing based on oxidation‐reduction reaction (e.g. gas, glutathione/disulfide bond).^[^ ^182^, ^183^, ^184^ ^]^ On the other hand, noncovalent, supramolecular binding interactions can also be used to capture analytes. For example, electrostatic interactions, hydrogen bonds,^[^ ^185^ ^]^ aromatic stacking,^[^ ^186^ ^]^ hydrophobic interactions,^[^ ^187^ ^]^ van der Waals interactions,^[^ ^188^ ^]^ etc., are also widely used in this respect.^[^ ^38^, ^117^ ^]^ The majority of noncovalent interactions are relatively weak and reversible, and their efficiency is often compromised by solvent interferences, particularly in water, which disrupt the effective hydrogen bonding between a receptor and its analyte. As a result, the recognition elements can be divided into two types that provide either mono‐specificity or poly‐specificity (**Figure** **2**). In terms of introducing chemical specificity to the binding site, one known approach relies on the use of antibodies, nucleic acids including aptamers, small peptides, and selective metal chelators to realize the “one‐to‐one/lock‐to‐key” recognition.^[^ ^189^, ^190^, ^191^ ^]^ For example, antibody,^[^ ^192^, ^193^ ^]^ aptamer,^[^ ^189^, ^194^, ^195^ ^]^ antimicrobial peptide,^[^ ^196^ ^]^ phage,^[^ ^197^, ^198^ ^]^ etc., are widely used in detecting bacteria with the help of nanomaterials,^[^ ^8^ ^]^ namely biosensor.^[^ ^40^, ^199^, ^200^ ^]^ These biological receptors are expensive and fragile. However, it is an ongoing challenge to produce synthetic receptors that interact with analytes in water through supramolecular interactions, with high specificity and sensitivity. On the other hand, the strategy of ‘one‐to‐more’ detection that utilizes various noncovalent interactions with analytes offers a powerful approach. For example, supramolecular host–guest interactions and multiplex detection are one of two famous applications based on this.^[^ ^104^, ^201^ ^]^ Future efforts and success in the development of such supramolecular receptors will be a crucial step in the development of versatile, robust, and cost‐effective sensors that can detect multiple analytes.^[^ ^202^, ^203^ ^]^

The molecular recognition process is a fundamental step in the design of nanosensors. It has long been assumed that ideal recognition elements should have high selectivity and strong binding affinity for a particular analyte, typically characterized by a 1:1 binding ratio between the receptor and the target analyte.^[^ ^73^, ^204^, ^205^ ^]^ The analyte can bind to the detection elements of the nanosensor through noncovalent interactions such as electrostatic interactions, hydrogen bonds, halogen bonds, dispersive interactions, cation or anion‐π interactions, and also through effects such as hydrophobic effect.^[^ ^10^, ^201^ ^]^ Noncovalent interactions between analytes and many synthetic receptors often exhibit a lower interaction strength in aqueous media. This is usually due to the high solvation of the analytes, which leads to effective shielding. For example, the analyte can form hydrogen bonds with water molecules, which can affect its ability to interact with the receptor. In this respect, the ongoing effort by the supramolecular sensing community is based on designing efficient synthetic receptors that maintain selectivity and the required binding affinity for substances in biofluids and food. Successful examples of the application of noncovalent interaction and analyte detection are the glucose sensors with molecular detection technology developed independently by Senseonics and GlySure Ltd. using fluorescent boronic acid probes,^[^ ^206^, ^207^ ^]^ or the cation‐selective chemosensors that led to the development of a supramolecular sensor cassette by OPTI Medical Systems, Inc.^[^ ^208^, ^209^ ^]^ Strong coordinative bonds by high affinity chelators, however, have been shown to retain a good affinity for the detection of metal ions.^[^ ^38^, ^210^, ^211^ ^]^ Recent research on synthetic receptors, including macrocycles and molecular tweezers, for the detection of analytes in aqueous media has been widely reviewed and the reader is referred to these excellent papers.^[^ ^38^, ^212^ ^]^ As representative macrocyclic compounds, cryptands,^[^ ^213^ ^]^ calix[n]arenes,^[^ ^214^, ^215^ ^]^ cavitands,^[^ ^216^, ^217^ ^]^ naphthotubes,^[^ ^218^, ^219^, ^220^ ^]^ and cucurbit[*n*]urils^[^ ^221^, ^222^, ^223^, ^224^ ^]^ can strongly bind biorelevant analyte classes, such as metabolites, neurotransmitters, steroids or metal cations in aqueous media. Additionally, molecularly imprinted polymers (MIP) are the rapid and direct determination of the interaction with analyte.^[^ ^225^, ^226^, ^227^, ^228^ ^]^ The MIP techniques are one of the most efficient and versatile methods for specific molecular recognition with a polymeric network, and have been used in gas, drugs, pesticides, and explosive chemicals sensing.^[^ ^229^, ^230^ ^]^ Among synthetic molecular receptors and antibody‐ and antigen‐based biological receptors commonly employed in established immunosensors,^[^ ^231^ ^]^ monoclonal antibodies are particularly favored. This preference stems from their superior specificity and affinity for antigens relative to other antibody classes.

This higher specificity and affinity often lead to binding affinities in the nanomolar to picomolar range.^[^ ^232^, ^233^, ^234^ ^]^ While antibodies can achieve high specificity and practically useful binding affinities, their disadvantage lies in their costly production and limited stability. Due to these challenges, researchers have continuously developed several alternative protein scaffolds with stable core structures, such as single‐chain variable fragments (scFv), antigen binding fragment (Fab), nanobody, and minibody.^[^ ^235^, ^236^ ^]^ Aptamers are short and single‐stranded RNA or DNA sequences, able to recognize and bind to non‐nucleic acid analytes, including small molecules, proteins, and pathogens with high affinity and specificity, which have been described in the last decades. High affinity aptamers are typically obtained thanks to the SELEX process or similar strategies.^[^ ^237^, ^238^, ^239^, ^240^ ^]^ Aptamers, in particular, are widely researched in sensing technologies when used as probes for proteins, toxins, small organic molecules, or metals, and are under development as potential drugs.^[^ ^241^, ^242^, ^243^, ^244^ ^]^ Enzymes, which are known to catalyze specific reactions with high efficiency, produce measurable signals in nanosensors and have been widely used as detection elements.^[^ ^245^, ^246^ ^]^ Enzyme sensors are capable of measuring either the catalysis or inhibition of enzymes in response to the presence of various analytes, including pesticides, glucose and hydrogen peroxide.^[^ ^247^, ^248^, ^249^ ^]^ In addition, phages that can specifically recognize pathogens are also used as detection elements.^[^ ^73^ ^]^ It is important to note that these biomolecules and organisms are generally less stable than chemical molecules.

Regarding small molecular probes, receptors that engage in covalent interactions with the analyte, and boronic acid are prominent examples that have been used to recognize saccharides through the formation of boronic esters with 1,2 diols.^[^ ^250^, ^251^ ^]^ Michael addition strategies that involve nucleophilic addition of a nucleophile or carbanion to an α,β‐unsaturated carbonyl group are widely used for biothiol detection, with squaraines, maleimide and acrylamide derivatives commonly used as Michael acceptors.^[^ ^252^, ^253^, ^254^ ^]^ Additionally, some chelating ligands can also interact with selected metal ions.^[^ ^255^ ^]^

### 3.2 Nanomaterials for Nanosensors

The IUPAC defines nanoparticles as objects having at least one external dimension in the range of 1–100 nm.^[^ ^256^ ^]^ However, several authors extend the term “nanoparticle” to particles larger than 100 nm (up to several hundred nanometers) as long as they exhibit different physicochemical properties than their bulk phase.^[^ ^257^ ^]^ Nanoparticles offer a wide range of functionalization options: They can be modified with multiple copies of identical probes and chemosensors. Furthermore, they can be co‐functionalized with additional components such as dyes, power enhancers, or cellular target molecules, making them effective colloidal carriers for water‐insoluble molecular probes and chemosensors.^[^ ^258^ ^]^ This approach offers several advantages.^[^ ^259^ ^]^ First, the small size of the nanoparticles leads to a large surface area relative to their mass, which facilitates efficient functionalization with a high density of recognition elements on their surface. Secondly, it is possible to electronically couple both the receptor and the receptor‐analyte complex to the nanoparticles. This coupling enables energy transfer processes, which can be leveraged for ratiometric detection. This effect is used in particular for plasmonic nanoparticles and optical nanosensors, but also for applications with electrochemical readout.

The role of nanoparticles in a nanosensor can be divided into two main types: passive and active sensing elements (**Table** **2**). Passive nanoparticles act as basic sensing components, usually carrying the receptors and reporter units required for detection and increasing the surface area and density of functional groups for detection only. In contrast, active nanoparticles play a direct role in signal transduction and actively participate in the detection process, for example, the plasmonic nanoparticles, which are typically used in SERS‐ and FRET‐based techniques where molecular species are detected. Over the past, the use of plasmonic nanomaterials and magnetic nanomaterials to modulate the signal prior transduction has been widely reported.^[^ ^260^, ^261^, ^262^, ^263^, ^264^, ^265^ ^]^ Plasmonic nanomaterials, such as gold or silver nanoparticles,^[^ ^266^, ^267^ ^]^ are widely used in the development of nanosensors,^[^ ^268^ ^]^ exhibiting plasmon oscillations with specific resonance wavelengths, which have been utilized for significant advances in ultrasensitive detection, in particular by SERS.^[^ ^260^, ^269^, ^270^, ^271^, ^272^, ^273^ ^]^ SPR, another important application, uses plasmonic nanomaterials to characterize molecular interactions and detect analytes. Their sensitivity is mainly influenced by changes in the local refractive index caused by interactions between target molecules and the sensor.^[^ ^274^, ^275^ ^]^ In addition, surface‐enhanced fluorescence (SEF) is a highly sensitive detection method that is also based on the properties of plasmonic nanomaterials.^[^ ^276^, ^277^, ^278^ ^]^ Aside from SERS‐active nanomaterials, fluorescent nanoparticles are extensively used for sensor applications and include a variety of nanoparticles such as carbon dots,^[^ ^279^, ^280^, ^281^ ^]^ quantum dots,^[^ ^282^, ^283^, ^284^ ^]^ polymer dots and nanoparticles,^[^ ^285^, ^286^ ^]^ fluorescent silica nanoparticles,^[^ ^287^, ^288^ ^]^ nanodiamonds,^[^ ^289^ ^]^ metal particles,^[^ ^290^, ^291^ ^]^ silicon,^[^ ^292^, ^293^ ^]^ upconverting nanoparticles.^[^ ^294^, ^295^ ^]^ Compared to organic fluorophores, nanoparticle‐based emitters generally exhibit higher chemical stability and better photophysical properties.^[^ ^296^, ^297^ ^]^ These properties include higher photoluminescent quantum yield and lower susceptibility to photobleaching. In addition, they are also suitable for advanced fluorescence applications such as fluorescence lifetime measurement and fluorescence resonance energy transfer.^[^ ^298^, ^299^, ^300^, ^301^, ^302^, ^303^ ^]^ Magnetic nanoparticles, especially the superparamagnetic nanoparticles, have excellent controllable properties, due to the strong magnetic responsiveness with external magnetic field. The magnetic nanosensors can be recovered from sample matrices and redispersed again by turning on/off the external magnetic field.^[^ ^265^, ^304^ ^]^ These characteristics can be used for analyte enrichment and separation, and sensing can also be realized by magnetic relaxation switching assay.^[^ ^67^, ^305^ ^]^ Silica‐based nanoparticles are also known as important nanomaterials that have significant applications such as sensing, drug delivery, and catalysis.^[^ ^306^, ^307^, ^308^, ^309^ ^]^ In addition to these typical nanomaterials, there are also other nanomaterials with multiple composition, e.g. MOFs, which can also be used in nanosensor.^[^ ^49^, ^310^, ^311^, ^312^ ^]^ Next, we will discuss the commonly used nanomaterials in detail.

**Table 2:** Examples of nanomaterials for the fabrication of nanosensors with multiplex detection.

Type of nanomaterials	Characters and Advantages	Type of sensor	nanoparticles	Sensing of application	Detection limit	Refs.
Plasmonic nanomaterials	Surface plasmon resonances, enhancement, and confinement of light	Local surface plasmon resonance nanosensor	Gold nanoparticles	Several pathogen DNA sequences	60 nM	[313, 314]
		SERS nanosensor	Gold nanoparticles	Alzheimer's disease(AD) core biomarkers (Aβ(1−42) oligomers and Tau protein)	3.7 × 10^−2^ nM, 4.2 × 10^−4^ pM	[315]
		Fluorescence‐enhancement sensor	Al nanodisks	insulin, vascular endothelial growth factor, and thrombin relevant for diabetes,	1 fM	[316]
		Scattering spectra sensor	Gold nanorod	Pb^2+^, Hg^2+^	5 nM, 1nM	[317]
Fluorescent nanomaterials	High brightness and photostability	Fluorescent sensor array	Carbon dots	Identification of six bacteria	100% accuracy	[318]
		Immunochromatographic assay	Quantum dots	Mycotoxins (OTA, FB1, ZEN)	5 nm/mL, 20 ng/mL, 10 ng/mL	[319]
		Immunochromatographic assay	Polymer dots	Lung cancer biomarkers [arcinoembryonic antigen (CEA) and cytokeratin 19 fragment (CYFRA 21‐1)]	0.12 ng/mL, 0.07 ng/mL	[320]
		Fluorescent sensor array	mesoporous silica nanoparticles (MSNs) conjugated with fluorescein	Seven foodborne pathogens (*Escherichia coli, Salmonella spp., Listeria spp., Shigella spp., Campylobacter spp., Clostridium spp., and Vibrio spp*)	20‐34 CFU/g	[321]
		Immunochromatographic assay	Aggregation‐induced emission nanoparticles	aflatoxin B1 and zearalenone	6.12 pg/mL 26 pg/mL	[322]
		Fluorescent imaging	Fluorescent nanodiamonds	HeLa cells, miRNA		[323]
Magnetic nanoparticles	Separation and enhancement of *T_2_*	Fluorescent sensor	Fe_3_O_4_ nanoparticles and gold nanoparticles	kanamycin, 17β‐estradiol, and Pb^2+^	1.76 × 10^−4^ nM, 1.18 × 10^−4^ nM, and.29 × 10^−4^ nM (lead ion)	[324]
		Magnetic sensor	Magnetic nanoparticles	S. enteritidis	50 CFU/mL	[325]
Mesoporous Silica nanoparticles	high surface area, tunable pore size, and extraordinary chemical stability, which supports many potential sensors	Fluorescent sensor array	Mesoporous silica nanoparticles, quantum dots	four heavy metal ions (Hg^2+^, Cu^2+^, Cr^3+^, and Ag^+^)	2.51 nmol/L, 5.15 nmol/L, 3.81 nmol/L, and 5.74 nmol/L	[326]
MOFs	High porosity, tunable physical and chemical properties	Electrochemical sensor	MOFs HKUST‐1	Pb^2+^, Cd^2+^	33 nM, 50 nM	[327]

Plasmonic nanomaterials consist of (precious) metals such as gold, silver, and copper, which exhibit localized surface plasmon resonance (LSPR) phenomena, the extent and properties of which depend on the composition, shape, and size of the nanoparticles.^[^ ^328^ ^]^ As a result of LSPR, the fluorescence of dyes and Raman signals can be million times enhanced.^[^ ^329^, ^330^ ^]^ Plasmonic nanomaterials can also be used as a light source, optical switches, microscopes, waveguides, and lithographic tools.^[^ ^71^, ^331^ ^]^

QDs (quantum dots) are semiconducting nanoparticles and display excellent photophysical and optical properties,^[^ ^47^, ^282^, ^283^, ^284^, ^332^ ^]^ such high fluorescence quantum yields and stability against photobleaching and degradation. QDs exhibit distinct absorption and luminescence properties that are tunable on the basis of their shape and size, a phenomenon resulting from the quantum confinement of electrons. A notable feature of QDs is their bright and narrow‐band emission, which enables precise spectral identification. Moreover, because of their broad absorption spectrum, QDs can be excited simultaneously at a single wavelength. This property is particularly advantageous for multiplex detection, as it enables the simultaneous excitation of multiple QDs, each of which clearly emits at different narrow wavelengths. This allows the use of multiple emission colors in a single test, greatly enhancing the ability to detect different analytes in complex mixtures. QDs are functionalized with specific receptors to construct nanosensors.^[^ ^47^, ^333^ ^]^ These sensors work on the principle that binding of an analyte to the receptor modulates the excited state of the QD, resulting in a change in the intensity of the emission, usually observed as quenching. In addition to this direct modulation, the system can be enhanced by the incorporation of a secondary reporter dye. This enables ratiometric detection,^[^ ^334^, ^335^ ^]^ where the fluorescence of the QDs remains constant, but the emission intensity or color of the reporter dye changes in response to interaction with the receptor bound to the analyte. In addition, this secondary reporter dye can participate in FRET processes with the QD. In such a setup, the emission of the QD is sensitized by the dye, and this emission change can be made dependent on the presence of an analyte in the vicinity of these reporter dyes. Therefore, the combination of QDs and secondary reporter dyes in nanosensors provides a versatile platform for the sensitive detection of various analytes with potential applications in numerous fields, including biomedical diagnostics and environmental monitoring.^[^ ^336^, ^337^, ^338^ ^]^

Single‐walled carbon nanotubes (SWCNTs) are a type of sp^2^‐hybridized carbon allotrope, similar to fullerenes.^[^ ^339^, ^340^, ^341^, ^342^, ^343^ ^]^ SWCNTs, which structurally resemble a monolayer of graphite rolled into a tubular shape, exhibit unique optical properties that distinguish them in the field of nanomaterials. These nanotubes are characterized by different absorption and fluorescence spectra depending on their chirality and are particularly effective as near‐infrared (NIR) emitters.^[^ ^344^, ^345^ ^]^ The transparency of NIR light for biological tissue makes it advantageous for biomedical detection and is less subject to scattering than visible light, which is ideal for detection in complex media. Strano et al. have further developed SWCNT sensing through “corona phase molecular recognition”,^[^ ^346^ ^]^ a method that modulates fluorescence by altering surface interactions and enables selective detection of analytes based on the surface composition‐dependent emission properties of SWCNTs.^[^ ^347^ ^]^

Carbon nanodots (CDs) are a type of fluorescent nanoparticle consisting mainly of carbon and usually less than 10 nm in size.^[^ ^348^, ^349^, ^350^ ^]^ CDs are characterized by their high stability, excellent water dispersibility, low toxicity, and optical properties comparable to QDs.^[^ ^351^, ^352^, ^353^ ^]^ However, it should be noted that the photoluminescent quantum yield of CDs, which is between 10 and 30%, is generally lower than that of QDs, which is above 50%. The presence of carbonyl groups on CDs facilitates their relatively easy functionalization and enables versatility in diverse fields.

MOFs are crystalline materials with micro‐ to mesoporous structures consisting of metal ion clusters connected by organic ligands.^[^ ^354^, ^355^ ^]^ MOFs incorporating lanthanides are promising candidates for the development of fluorescence‐based sensors, as they exhibit remarkable stability and their light emission is highly monochromatic.^[^ ^49^, ^356^ ^]^ MOFs possess high microporosity and–in the case of hierarchical MOFs–mesoporosity, which are ideal for adsorbing analytes.^[^ ^357^, ^358^ ^]^ This adsorption is driven through interactions with the organic components lining the pore walls or with the metal nodes within the scaffold. When these organic components, which can be fluorescent, interact with an analyte, they change the optical properties of the MOF.^[^ ^359^, ^360^ ^]^ This change facilitates the development of optical sensors. MOFs can be synthesized in different sizes and shapes, and they can be easily doped with different elements and organic functions.^[^ ^361^ ^]^ This versatility is achieved by choosing from a wide range of available metal‐based junctions and organic linkers. Despite these advantages, a major challenge is to create MOFs that are stable in water. This aspect remains a major hurdle to the wider application of MOFs in different environments.^[^ ^362^ ^]^

Nanoparticles based on (mesoporous)silica and organosilica offer a more stable alternative to other nanomaterials and can be easily modified with organic functionalities.^[^ ^363^, ^364^, ^365^, ^366^ ^]^ (Organo)silica‐based mesoporous nanoparticles are generally nontoxic and can be produced relatively easily by sol‐gel chemistry in various sizes and shapes,^[^ ^367^, ^368^, ^369^, ^370^, ^371^ ^]^ whereby the pore surfaces and the outer surface of the particles can be functionalized by alkoxysilane chemistry.^[^ ^372^, ^373^ ^]^ Due to their improved stability and ease of functionalization, silica and organosilica nanoparticles are widely used for the development of nanosensors.^[^ ^287^, ^306^, ^308^, ^374^ ^]^

Magnetic nanomaterials are a class of materials exhibiting tunable nanoscale magnetism based on their compositions, structures, shapes, and functionalities.^[^ ^375^, ^376^, ^377^, ^378^, ^379^, ^380^, ^381^, ^382^ ^]^ Iron, cobalt, nickel‐based ferromagnetic elemental, oxide, alloy, or compositions are widely studied and display great potential in diagnosis, therapeutic, data storage, and catalysis.^[^ ^304^, ^383^ ^]^ Magnetic nanoparticles (MNP) can overcome length mismatch in the sensing process, shortening the distance and time of analytes to find the detector by collecting and concentrating the analytes.^[^ ^380^, ^381^, ^382^ ^]^ Hence, there are a large number of ultrasensitive sensor chosen MNPs as a component in sensing.^[^ ^379^, ^384^, ^385^ ^]^ For example, hybrid nanomaterials have great development as their superior combinational properties to a single nanomaterials, for example, Fe_3_O_4_ nanoparticles as core to form Fe_3_O_4_@MOFs, Fe_3_O_4_@MIP and Fe_3_O_4_@COFs materials.^[^ ^15^, ^310^ ^]^ The diffusion of water protons in the inhomogeneous magnetic field leads to relaxation of protons, resulting in concomitant change of relaxation time, especially the *T_2_*, which has a famous application of *T_2_*‐wighted magnetic resonance images.^[^ ^386^, ^387^ ^]^ In this, superparamagnetic particles gain more popularity because of each vectorized particle suffers an enormous magnetic moment, therefore, being widely used in MRSw.^[^ ^36^, ^67^, ^72^, ^388^ ^]^

Soft nanoparticles, such as fluorescently labeled surfactant micelles, have attracted a great deal of attention in the development of nanosensors.^[^ ^389^, ^390^ ^]^ Micelles, which are formed by the self‐assembly of surfactant molecules, usually have a spherical structure with a hydrophobic core and a hydrophilic shell in an aqueous environment. This spontaneous formation is driven by the balance between entropy and enthalpy. In water, micelle formation is primarily driven by the hydrophobic effect, although the process is thermodynamically unfavorable, both in terms of enthalpy and entropy of the system. Furthermore, micelles are dynamic entities capable of exchanging monomers with the surrounding solution and can adsorb analytes either at their water interface or in their lipophilic cores.^[^ ^391^ ^]^ These are some nanoparticles that have been extensively studied, but other classes of nanoparticles also play important roles in the materials field, such as carbon‐based nanomaterials, polymer‐based nanomaterials, and other metallic nanoparticles; they all have excellent performance in sensing applications.^[^ ^8^, ^312^, ^392^ ^]^

Encoded micrometer/nanomaterials play a role as barcodes that include different elements such as optical, magnetic, shapes, and sizes, providing more readable tags than other kinds of nanomaterials. Each encoded particle has a specific code. These kinds of nanomaterials have great applications in multiplex analysis.^[^ ^120^, ^121^, ^393^, ^394^, ^395^ ^]^ The Luminex xMAP technology is a successful commercial example for multiplex immunoassay using dye encoded microbeads.^[^ ^396^ ^]^ Nanobarcodes are smaller in size and are more suitable to detect smaller analytes. The nanostructure acts as a nanocarrier with unique signatures, where the encoding elements can be self‐assembled, decorated on the surface, or embedded inside.^[^ ^20^, ^395^, ^397^ ^]^ Generally, the nano‐barcodes are easy to prepare and have large coding capacity with various shapes, beads,^[^ ^20^ ^]^ wires,^[^ ^398^ ^]^ rods,^[^ ^399^ ^]^ disks,^[^ ^400^ ^]^ tubes,^[^ ^401^ ^]^ etc. Additionally, bio‐barcodes also can be added to this category.^[^ ^402^, ^403^ ^]^ Encoding techniques determine how to decode the signals originating from these nano‐barcodes, of which optical encoding is the most popular, varying from fluorescence, Raman, and reflection to lifetime.^[^ ^404^ ^]^ There are also other kinds, like shape‐based materials, and electrochemical signal‐based materials.^[^ ^405^, ^406^ ^]^ For example, nanobarcodes with SERS coding elements need Raman measurement, while nanobarcodes with fluorescent elements can be identified by fluorescence microscopy.^[^ ^407^, ^408^ ^]^ Nano‐barcodes solved many problems in multiplex assay. Firstly, they are capable of generating numerous distinguishable signals, each of which can be detected by instruments. The degree of differentiation between these signals directly influences the speed, accuracy, and resolution of detection, determining whether these signals can be effectively used and identified in the presence of multiple targets. With a greater number of distinct signals, enhanced detection performance is achieved. Secondly, they enhance the signal‐to‐noise ratio and enable simultaneous detection, providing solutions for complex samples with various target conventions. Lastly, they streamline the detection procedures.

### 3.3 Multiplex Assay Achieved Through Multiple Single Detection Reactions Within a Single System

**Figure 3:**
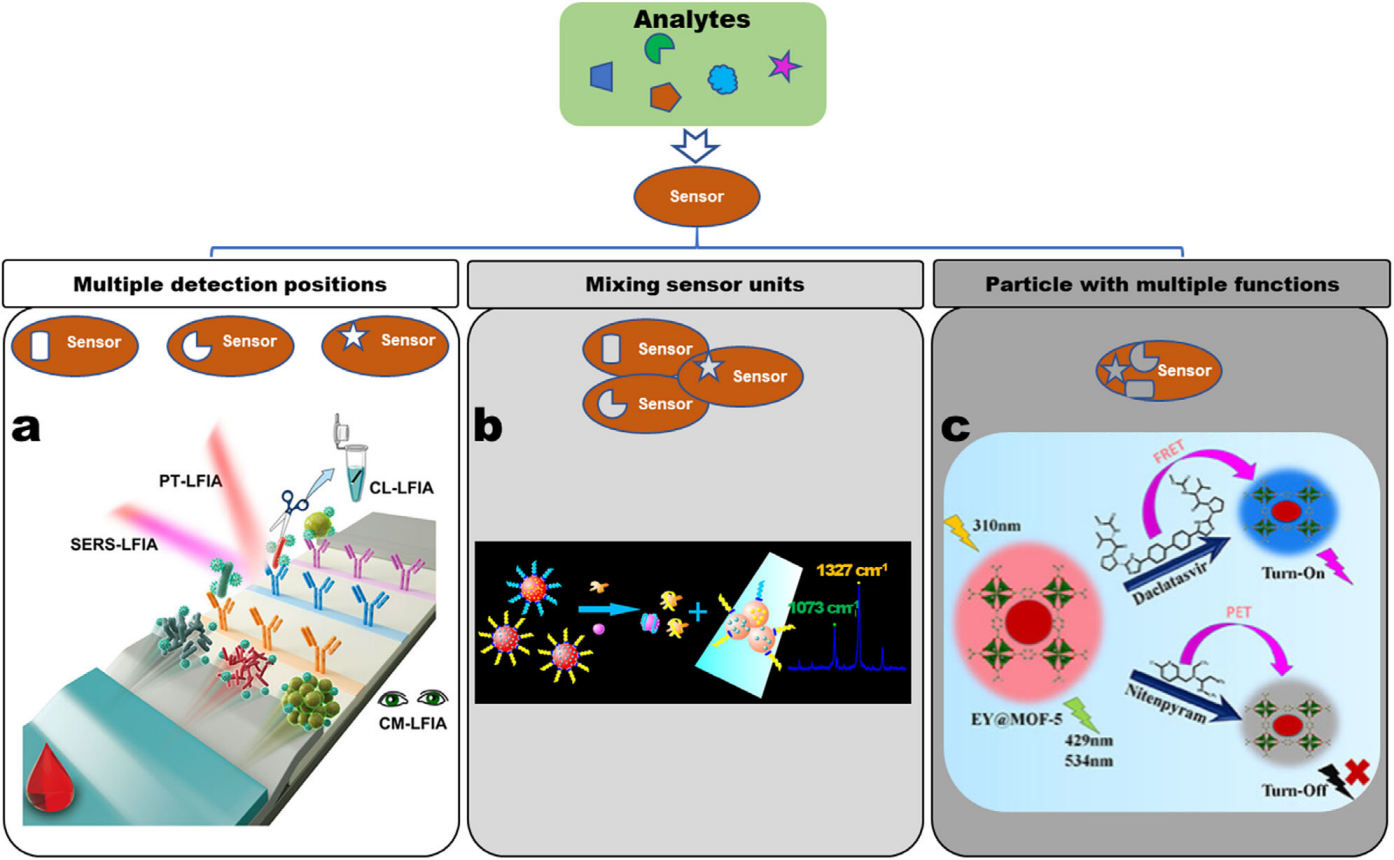
Strategies for achieving multiplex detection by concentrating multiple single detection reactions. Detailed approaches include: a) increasing the number of detection sites, b) combining sensor units within a single detection process, and c) designing multifunctional nanosensors. Reproduced with permission.^[^ ^315^ ^]^ Copyright 2019, American Chemistry Society. Reproduced with permission.^[^ ^414^ ^]^ Copyright 2024, American Chemistry Society. Reproduced with permission.^[^ ^415^ ^]^ Copyright 2023, American Chemistry Society.

The single detection reaction is based on the “lock and key” strategy.^[^ ^409^, ^410^, ^411^ ^]^ If many assays are required in one sample, an easy and effective approach is to combine multiple single detection reactions (**Figure** **3**), for example, based on an array, lateral flow assay, or electrochemical sensor.^[^ ^313^, ^412^, ^413^ ^]^ In this approach, each particle with a specific binding element functions as an independent sensor unit. To streamline the detection procedures, uniform nano‐substrates are employed, and signals are collected using consistent methods. This strategy is applied in various sensing applications. One approach involves centralizing multiple detection sites within an array, allowing for step‐by‐step signal detection at each point (Figure 3a). For instance, in detecting non‐small cell lung cancer‐associated exosomal miRNAs, gold nanoparticles and silver nanocubes were utilized to fabricate distinct probes by varying the capture DNA. The analysis was conducted using surface plasmon resonance imaging (SPRi), and a multiplex assay was achieved by incorporating four detection sites into an SPRi chip.^[^ ^314^ ^]^ Second, multiplex detection can be achieved in a single step using mixed probes by combining the sensor units (Figure 3b). Third, advancements in synthetic techniques enable multiple detection reactions within a single particle (Figure 3c). This approach simplifies sensor fabrication and reduces costs. For example, the composite of eosin Y and MOF‐5 (EY@MOF‐5) exhibits dual‐emission fluorescence. In the presence of daclatasvir and nitenpyram, the fluorescence of EY@MOF‐5 shows an increase and decrease in response, respectively.^[^ ^414^ ^]^ In this strategy, the capacity for multiplex detection is limited. Increasing the number of sensor units necessitates generating a greater number of distinct signals and identifying additional corresponding binding elements, which complicates the synthesis of multifunctional nanoparticles.

### 3.4 Multiplex Assay Utilizing Micron/Nano‐Barcodes

Barcoded nanoprobes or microspheres, each carrying a unique signal code, are becoming increasingly popular for performing multiplex assays^[^ ^393^, ^416^ ^]^ In this platform, a detection system comprises several different probes, with each probe corresponding to a specific piece of information or analyte. This includes the quantification or identification of different analytes. The barcode test is a stand‐alone labeling method that integrates well with conventional analytical chemistry techniques. These different probes together form a comprehensive barcode library enabling high‐throughput measurements and efficient sample processing. This method enables the analysis of numerous analytes with a single toolkit and supports the simultaneous processing of multiple samples in a single batch, a feature that is essential for the detection of a wide range of contaminants, particularly in food safety.

**Figure 4:**
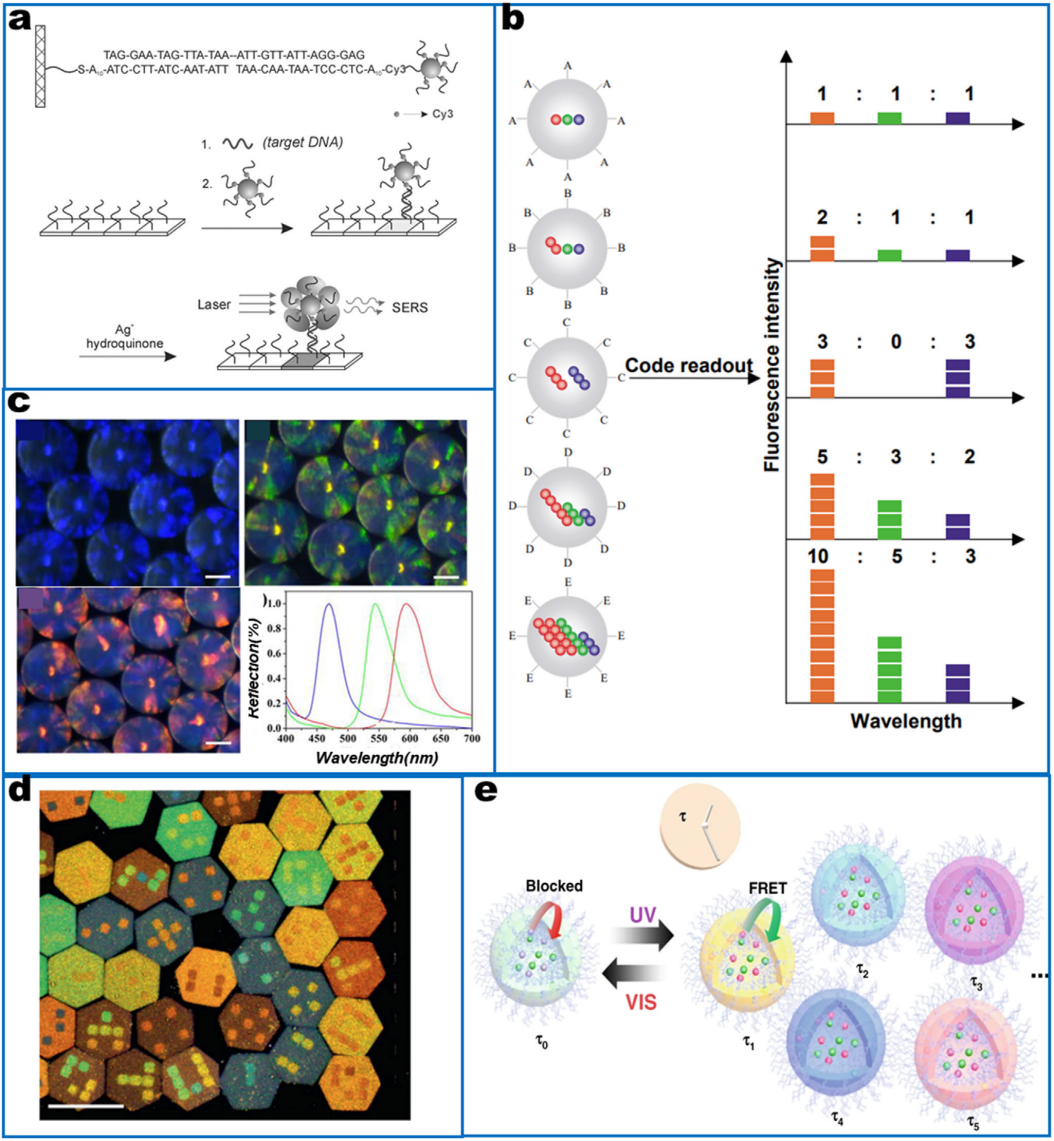
Optical encoding based on particles for multiplex detection. a) SERS encoding is based on gold nanoparticles by changing the Raman molecules. Reproduced with permission.^[^ ^270^ ^]^ Copyright 2002, Science (AAAS). b) Fluorescent encoding based on QDs with different wavelengths and intensity. Reproduced with permission.^[^ ^395^ ^]^ Copyright 2001, Springer Nature. c) Photonic crystal (PhC) barcodes with different reflection intensities and scale bars of 100 µm.^[^ ^134^ ^]^ Copyright 2020, American Chemistry Society. d) Graphical encoding with particles with tunable structural colors. Reproduced with permission.^[^ ^121^ ^]^ Copyright 2010, Springer Nature. e) Fluorescence lifetime encoding based on nanogels through emulsion polymerization. Reproduced with permission.^[^ ^421^ ^]^ Copyright 2020, Springer Nature.

Barcode strategies have evolved using a variety of building blocks, ranging from chemical molecular tags to nanostructures at the nanoscale. To date, a variety of encoding strategies have been developed that include graphical, physical, optical, electronic, magnetic, and chemical encodings. These methods have led to the development of numerous precise codes for multiplex recognition.^[^ ^119^, ^121^, ^393^, ^395^, ^405^, ^417^, ^418^, ^419^ ^]^ Among these multiplex detection methods, optical readout is superior in terms of the simplicity of the encoding and decoding processes (e.g., by different emission colors) (**Figure** **4**), large multiplex capacity, and miniaturization of detection devices, and is considered the most flexible and convenient strategy.^[^ ^420^ ^]^ In addition to the emission color, which can be used for coding, other optical properties of nanosensors can also be used, including emission intensity, scattering signals, and lifetime.^[^ ^394^, ^404^, ^418^, ^421^ ^]^ The encoded nanoparticles provide multiple signal outputs, and oftentimes, the sensing was realized by labeling related recognition units to each kind of particle.^[^ ^422^ ^]^ Although these barcoded nanomaterials enlarge the encoding capacity, the sensing capacity is still limited by the recognition elements, as the challenge of finding highly specific and sensitive binding units in quantities sufficient.

The bio barcode assay was first proposed by Mirkin et al. for the detection of protein.^[^ ^174^, ^402^, ^403^ ^]^ This is an ultrasensitive nanoparticles amplification analyzing system, normally, two types of particles were used (magnetic and gold nanoparticles), and the bio barcodes (a fragment of DNA sequence without recognition function to analyte) were labeled on gold nanoparticles, in the presence of a target a sandwich structure was formed. As a large amount of DNA was detached from gold nanoparticles, by varying the sequence of DNA fragments, sensitive and multiplex detection was realized indirectly.^[^ ^423^, ^424^, ^425^ ^]^ Here, signal amplification detection methods mainly include fluorescence labeling methods,^[^ ^426^, ^427^, ^428^ ^]^ colorimetric methods,^[^ ^429^, ^430^ ^]^ electrical methods,^[^ ^431^ ^]^ and immune PCR methods.^[^ ^432^, ^433^ ^]^ The bio barcoded assay has high specificity and sensitivity, having been widely applied in food safety testing, medical diagnosis, and environmental monitoring.^[^ ^434^ ^]^

In the barcode‐based multiplex assay, each code corresponds to a sensor unit, the multiplex detection was mainly realized by setting multiple detection positions.^[^ ^416^ ^]^ Compared to the strategy outlined in section 3.2, micrometer/nanobarcodes offer a significantly greater number of distinct signals. However, the assay remains constrained by the limited availability of binding elements. Additionally, most encoding strategies rely on optical encoding, which involves mixing optical precursors in varying ratios. This approach poses challenges in accurately controlling the composition of the barcodes.

### 3.5 Logic Gates in Multiplex Detection Applications

**Figure 5:**
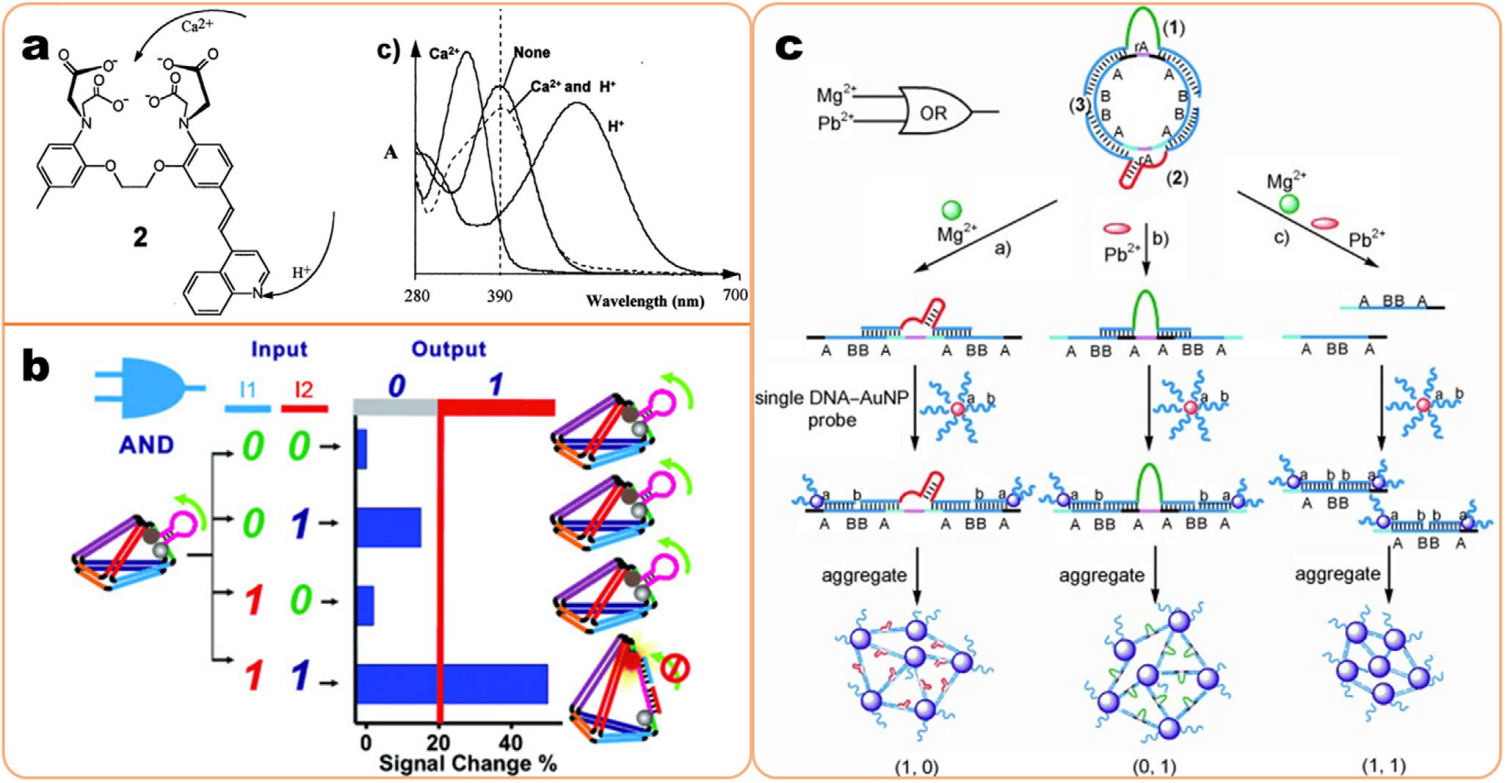
Representative examples of multiplex detection based on logic gates. a) molecular structures of “AND” and “XOR” logic gates for Ca^2+^ and ^H+^ sensing. Reproduced with permission.^[^ ^446^ ^]^ Copyright 2000, American Chemical Society. b) “AND” logic gate with Hg^2+^ and H^+^ ions as inputs based on 3D DNA tetrahedra. Reproduced with permission.^[^ ^454^ ^]^ Copyright 2012, John Wiley and Sons. c) “OR” logic gate system activated by Mg^2+^ and Pb^2+^ using gold nanoparticles and DNA composite. Reproduced with permission.^[^ ^455^ ^]^ Copyright 2010, John Wiley and Sons.

A logic gate is a type of Boolean logic application device that uses ‘1’ to represent ‘Yes’ and ‘0’ to represent “no”.^[^ ^435^, ^436^ ^]^ Typically, nanosensors provide quantitative information with targets. But in practical applications, especially for fast detection with many samples, a qualitative and yes/no answer is significant for the end user in terms of the presence/presence of targets.^[^ ^437^ ^]^ Nanosensors can be engineered in conjunction with logic gates, where the presence of a specific analyte is designated as the ‘1’ value (Boolean TRUE), and the absence of the target is specified as the ‘0’ value (Boolean FALSE). Using the presence of the analyte as an input, the logic gates can be categorized into single‐input logic gates (“YES” and “NOT” logic gates), two‐input logic gates (“OR”, “AND”, “XOR”, “INHIBIT”, “NOR”, “NAND”, “XNOR”, and “IMPLICATION” logic gates), and multiple‐input logic gates.^[^ ^438^, ^439^, ^440^, ^441^, ^442^ ^]^ Small molecules and nucleic acids are commonly employed in the construction of logic gates (**Figure** **5**).^[^ ^441^, ^443^, ^444^, ^445^ ^]^ Fluorescent molecules undergo significant changes in their electromagnetic and photophysical properties in response metal ions, resulting in “on” and “off” states. These states can be represented as “1” and “0”, demonstrating their potential for logic operations (Figure 5a).^[^ ^441^, ^446^ ^]^

In comparison, nucleic acids are programmable (Figure 5b), offering broader applications, targeting various entities such as proteins, small molecules, and whole cells.^[^ ^447^, ^448^, ^449^ ^]^ Additionally, nucleic acids can be easily modified with various molecules or moieties, including enzymes, antibodies, fluorescent molecules, and metal nanoparticles (Figure 5c). Numerous DNA‐based sensors have been designed in conjunction with logic gates.^[^ ^444^, ^450^, ^451^ ^]^ Simple logic gates, such as “OR” and “AND,” can be integrated into a single nanosensor; for instance, these gates have been used to detect Mg^2+^ and UO_2_^2+^.^[^ ^222^ ^]^ In some cases, simultaneous detection of multiple targets is achieved by incorporating the sensing units into one platform, allowing for signal readout in the presence of all targets.^[^ ^452^, ^453^ ^]^

### 3.6 Sensor Array

**Figure 6:**
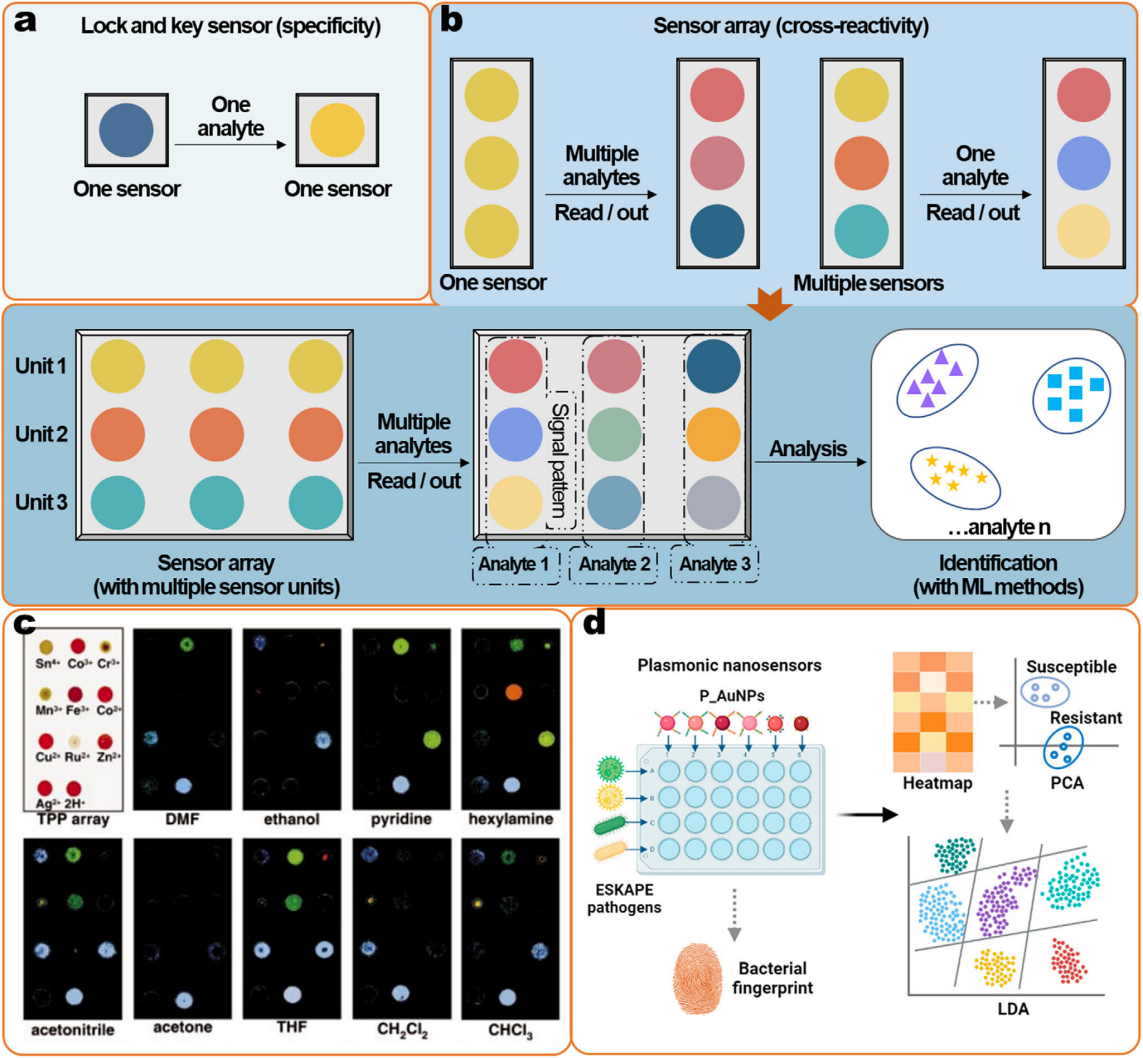
a) Illustration of a traditional nanosensor with high specificity to an analyte, producing a single response. b) Sensor array comprising a set of cross‐reactive sensor units, generating multiple responses for each analyte and providing fingerprint‐pattern recognition. Representative examples of multiplex detection based on sensor arrays. c) A colorimetric sensor array for odor visualization utilizing metalloporphyrins as sensor units, with TPP representing 5,10,15,20‐tetraphenylporphyrinate(‐2). Reproduced with permission.^[^ ^42^ ^]^ Copyright 2000, Springer Nature. d) Identification of antibiotic resistance in ESKAPE pathogens using a sensor array composed of plasmonic nanosensors, facilitated by machine learning. Reproduced with permission.^[^ ^138^ ^]^ Copyright 2023, American Chemical Society. ESKAPE is an acronym for six pathogens: *Enterococcus faecium, Staphylococcus aureus, Klebsiella pneumoniae, Acinetobacter baumannii, Pseudomonas aeruginosa, and Enterobacter spp*.

In contrast to the 1:1 binding scenario, in which specific receptors detect analytes with high selectivity, a sensor array identifies an analyte through the combined signals of several less selective receptors. This method mimics the gustatory and olfactory systems and relies on the overall response pattern of the array rather than highly specific interactions between the individual analytes and receptors (**Figure** **6**).^[^ ^173^, ^456^, ^457^ ^]^ The receptors used in this method have low selectivity, that is, each receptor can recognize several analytes, albeit to varying degrees. This property leads to increased cross‐reactivity. By using multiple different non‐selective receptors, a unique signal pattern is generated. This pattern serves as a characteristic “signal fingerprint” for each analyte and allows it to be distinguished (Figure 6b). For example, metalloporphyrins have open coordination sites for ligation, producing large spectral shifts and intense coloration, which can react with various chemicals and analytes.^[^ ^458^, ^459^ ^]^ A. Rakow utilized different metallated tetraphenylporphyrins deposited on reverse phase silica thin‐layer chromatography plates to create a colorimetric sensor array. Upon ligand binding with organic vapors, a series of color changes occurred, enabling optical chemical sensing (Figure 6c).^[^ ^42^ ^]^ This sensor array can discriminate subtle differences among various analytes. For example, it can distinguish different bacterial species with high specificity.^[^ ^460^ ^]^ Additionally, it can identify bacteria of the same species with different phenotypes. Furthermore, Yu et al. successfully used an SPR‐sensor array based on peptide‐functionalized gold nanoparticles to distinguish antibiotic resistance in ESKAPE pathogens (Figure 6d).^[^ ^138^ ^]^

Often, the signals from sensor arrays are complex and chaotic, necessitating pretreatment with suitable methods and the selection of appropriate pattern recognition techniques for effective processing and analysis.^[^ ^461^, ^462^ ^]^

Chemometric methods are employed to analyze these signals. For instance, pattern recognition protocols like PCA and linear discriminant analysis (LDA) are extensively used.^[^ ^463^ ^]^ Both LDA and PCA utilize matrix techniques to decompose the raw data and produce score plots, which indicate the level of discrimination. Although the mathematics behind LDA and PCA can be complex, chemists can easily assess the discrimination by examining the score plots. Such plots will show a clear separation between different analyte classes and tight clustering within the same analyte class.

Increasing the number of recognition units enhances precision and the ability to detect a broader range of analytes. However, this also increases the data dimensionality, necessitating the use of advanced machine learning algorithms such as Random Forest (RF), t‐distributed stochastic neighbor embedding (t‐SNE), and convolutional neural networks (CNN). While PCA has low computational overhead and does not require parameter tuning, RF can handle many parameters and rank variables effectively, and t‐SNE can visualize high‐dimensional data. These algorithms typically offer higher classification accuracy than PCA. For instance, a plasmonic sensor array composed of six types of peptide‐functionalized gold nanoparticles (each with four duplicates) was used to identify 12 antibiotic‐resistant ESKAPE pathogens. This identification was based on the various interactions between the pathogens and peptides, measured by the variation in maximum wavelength (∆λ) in the surface plasmon spectrum.^[^ ^138^ ^]^ Here, each type had a fingerprint, there ML algorithms were used to analyze the bacterial fingerprint, including PCA+HCA, RF, and t‐SNE+LDA. Each target had a unique fingerprint, and machine learning algorithms such as PCA combined with hierarchical cluster analysis (PCA+HCA), RF, and t‐SNE combined with linear discriminant analysis (t‐SNE+LDA) were employed to analyze these bacterial fingerprints.

SERS spectra provide unique information about analytes, eliminating the need to encode SERS signals like traditional sensor arrays. This is particularly advantageous for detecting analytes with similar structures. Interestingly, SERS techniques have facilitated the development of another type of sensor array. By integrating programmable units (U_A_, U_B_, U_C_) into a single chip, various gases can be detected.^[^ ^464^ ^]^ Each unit operates through physisorption or chemisorption and employs different SERS‐based detection techniques. For example, U_A_ can detect physically absorbed aromatic compounds with label‐free SERS signals, U_B_ is functionalized with 2,4‐dinitrophenylhydrazine to react with aldehydes or ketones, and UC can detect hydrogen sulfide through the decreased SERS intensity of a Raman reporter. A similar strategy was used to detect multiple cytokine biomarkers using plasmonic chips functionalized with antibodies.^[^ ^465^ ^]^

### 3.7 Label‐Free Strategy with SERS Spectrum

Label‐free SERS provides rich spectroscopic information for the identification of analytes without the need for tags.^[^ ^466^, ^467^, ^468^ ^]^ With the assistance of ML and multivariate calibration, Raman spectroscopy analysis becomes feasible in complex matrices.^[^ ^469^, ^470^ ^]^ This is particularly valuable for analyzing samples involving strong molecular interactions, such as bacterial drug resistance, diagnostics, and the quality control of edible oils.^[^ ^471^, ^472^, ^473^ ^]^ For example, a SERS substrate composed of Ag‐coated Si nanowires, which feature a 3D surface, increased the contact area with bacteria, leading to higher sensitivity in detecting antibiotic‐resistant strains.^[^ ^474^ ^]^ A Siamese neural network model was employed to classify the Raman spectra from 12 different bacterial species. Luis M. Liz‐Marzán's research group developed a microfluidic chip that accelerates the collection of cell supernatants and the acquisition of their respective SERS spectra. Supported by a SVM architecture, this system can discriminate the diversity of the secretum of dying cells.^[^ ^475^ ^]^ This setup was used to observe multiple stress conditions in cell cultures as a proof of concept.

In this strategy, the SERS substrate plays a crucial role due to its higher enhancement capabilities, which result in more characteristic peaks and greater accuracy. Researchers are continuously seeking more efficient substrates for sample preparation and sensitive signal acquisition. ML algorithms significantly facilitate data analysis; depending on the dimensionality, various algorithms are employed to achieve high accuracy. However, two challenges remain: the performance of ML algorithms depends on the size of the dataset, requiring a substantial training set that incurs higher experimental costs.^[^ ^476^ ^]^ Additionally, the validity of the trained model relies on correctly learned features, but due to the “black box” nature of these models, the accuracy of prediction is often lower than the training results.^[^ ^477^ ^]^ These issues necessitate innovation in algorithm development. To address these challenges, Tian's group designed a novel algorithm‐based framework that requires fewer experiments and provides visualization of the algorithm. In detecting polycyclic aromatic hydrocarbons, this approach increased sensitivity by at least an order of magnitude and achieved accuracy higher than 99%.^[^ ^478^ ^]^

### 3.8 The Perspective of ML

In the preceding sections, we discussed how ML algorithms facilitate data analysis across various strategies. This section provides a general overview of ML, covering the types of ML algorithms (**Table** **3**), the necessity of ML algorithms, and their future prospects. For more detailed information, readers are referred to other comprehensive works on the subject.^[^ ^479^, ^480^, ^481^ ^]^ ML algorithms are sophisticated programs that learn patterns from existing data using logic and mathematics. They extract features from complex output signals and are instrumental in prediction, classification, and clustering, thereby enhancing the accuracy of nanosensor detection. Based on their learning methods, ML algorithms are broadly categorized into three types: supervised algorithms, unsupervised algorithms, and reinforcement learning. These algorithms find extensive applications in fields such as food safety, environmental monitoring, medicine, and beyond.^[^ ^482^, ^483^ ^]^

1. Supervised Algorithms: These algorithms learn a function using labeled datasets to train models, making them well‐suited for classifying data or predicting outcomes accurately. Common supervised algorithms include neural networks, naive Bayes, linear regression, logistic regression, support vector machines, K‐nearest neighbor, and random forest.
2. Unsupervised Algorithms: Unlike supervised algorithms, unsupervised algorithms use unlabeled datasets to discover patterns that aid in dimensional reduction, clustering, and visualization. This is particularly useful for addressing uncertainty regarding common properties within a dataset. Common unsupervised algorithms include hierarchical clustering, Gaussian mixture models, and K‐means. Semi‐supervised algorithms, which require labels for only a small portion of the data, also fall into this category, effectively minimizing time and cost in obtaining training datasets.
3. Reinforcement Learning: This type of algorithm involves an agent that interacts with an environment and takes actions to minimize risk or maximize reward based on a performance metric. The agent continuously learns until the full range of possible states is explored. Reinforcement learning is highly effective in optimization tasks, such as searching for maximal SERS enhancement. Unlike supervised algorithms, the agent autonomously determines the ideal behavior to maximize performance, requiring only a reinforcement signal as a success metric. Common reinforcement learning algorithms include Q‐learning, temporal difference, and deep adversarial networks.

**Table 3:** Brief description of typical ML algorithms.

Name	Function	Description
Principal component analysis (PCA)	Dimensionally reduction	PCA extracts a few important indicators in the dataset with multiple indicators
Linear Discriminant Analysis (LDA)	Dimensionally reduction	LDA provides a projection of a training dataset that best separates the examples by their assigned class
Random Forest (RF)	Decision trees	RF combines the output of multiple trees to reach a single result for classification, regression, etc.
Support Vector Machine (SVM)	Binary classification of data	SVM finds a hyperplane in an N‐dimensional space (N‐the number of features) that distinctly classifies the data points.
K‐nearest Neighbors (KNN)	Classification and regression of data	KNN classifies a new given unseen data point by looking at the K given data points in the training set that is closest to it in the input or feature space.
Convolutional Neural Network (CNN)	Representation learning	CNN is a class of deep neural networks, including convolutional computation and a deep structure.
Partial Least Squares regression (PLS)	Reducing variables	PLS is a regression method based on covariance

How to choose the right approach for your data analysis? This depends on the structure and volume of data, and the use case. First, it is elementary to evaluate the input data and ensure it is labeled or not. Second, it is important to know if the researcher wants to solve a well‐defined problem or predict new outcomes. Lastly, it is necessary to check whether the algorithms have the required dimensionality (number of features, characteristics, or attributes) and can support the existing data volume and structure. ML is ubiquitous, but a few pitfalls and drawbacks also appear. It is difficult to incorporate traditional knowledge or theories into ML or to monitor its success. Despite these limitations, ML represents a paradigm shift in science, as it can discover hidden relationships in the data and provide better, faster, and more holistic solutions to unsolved questions.

Nanosensor techniques have proven to be outstanding tools in the fields of food contaminants detection and screening, characterized by their high throughput, exceptional sensitivity, and rapid detection capabilities. However, the increasing complexity of data structures, involving vast, high‐dimensional datasets, presents a significant challenge to traditional manual analysis methods. Fortunately, ML models can extract deep features from bio‐signals or complex relationships between inputs and outputs through appropriate multivariate modeling, thereby enabling effective processing, classification, and prediction. The main purposes of preprocessing the signals acquired from the nanosensor are as follows: 1) Mitigating Interferences: This involves addressing interferences caused by human actions, instrument limitations, environmental conditions, and other factors. Various techniques are applied to achieve this, including: (i) Polynomial baseline correction and the adaptive smoothness parameter penalized least squares method to rectify the baseline of the infrared spectrum.^[^ ^484^ ^]^ (ii) The Savitsky–Golay smoothing method to eliminate noise from the Raman spectrum,^[^ ^485^ ^]^ and (iii) Deep learning methods, such as cascaded convolutional neural networks and U‐Net, that perform noise removal and baseline correction.^[^ ^486^, ^487^ ^]^ 2) Enhancing Model Performance through Feature Engineering: Techniques such as PCA or t‐SNE are employed to map the raw surface‐enhanced Raman spectrum data into a new variable space, achieving data simplification and feature dimensionality reduction.^[^ ^488^ ^]^ 3) Optimizing, Expanding, and Enhancing Data: For instance, a generative adversarial network (GAN) consisting of a generator and a discriminator is utilized to determine various inverse metasurface designs based on the category, shape, and material of the target absorption spectrum.^[^ ^489^ ^]^

The ML methods employed in regression analysis encompass: 1) Linear Regression: This includes partial least squares (PLS) regression, which extracts principal components to address multicollinearity, and multiple linear regression (MLR), which considers the influence of multiple independent variables on the response variable. These methods find widespread applications in the multivariate calibration analysis of nanosensors.^[^ ^163^ ^]^ Techniques like elastic net and least absolute shrinkage and selection operator (LASSO) regression introduce penalty terms to combat overfitting.^[^ ^490^ ^]^ While these methods perform effectively for simple datasets, they exhibit limitations when confronted with high‐dimensional and nonlinear data. 2) Nonlinear Regression: Gaussian process regression (GPR), with its flexible covariance parameterization, is adept at handling both linear and nonlinear data, establishing high‐performance spectral correction models.^[^ ^491^ ^]^ However, it is crucial to exercise caution when configuring parameters for nonlinear regression methods to prevent excessive model complexity.

In the realm of common classification methods, we find the following approaches: (i) partial least squares‐discriminant analysis (PLS‐DA), which leverages surface‐enhanced Raman spectroscopy technology for the precise identification of COVID‐19.^[^ ^492^ ^]^ (ii) Logistic regression explores the correlation between composition and morphology using specific nanomaterials and research reagents.^[^ ^493^ ^]^ (iii) Additionally, a distinctive category of classification methods known as cluster analysis operates independently of sample labels.^[^ ^494^ ^]^ These methods classify samples by identifying similarities within the original data. Notable examples include hierarchical cluster analysis (HCA) and K‐means clustering.^[^ ^495^ ^]^

It is worth emphasizing that numerous methods are applicable to both regression analysis and classification. For example, ensemble learning techniques grounded in tree models have been employed, such as using random forests to predict the porosity of MOFs.^[^ ^496^ ^]^ Additionally, extreme gradient boosting (XGBoost) has been utilized to predict the neurodevelopmental age of rats with an impressive accuracy of 86.64%.^[^ ^497^ ^]^ Furthermore, methods based on neural networks have demonstrated their utility. For instance, artificial neural networks (ANN) have been used to inversely generate target spectra for eight‐shell nanoparticles.^[^ ^498^ ^]^ Moreover, CNN has been effectively combined with SERS spectra to detect seven different analytes.^[^ ^499^ ^]^

## 4 The Applications of Nanosensors for Multiplex Detection in Foods

### 4.1 Metal Ions

Heavy metal ions such as Pb^2^⁺, Cu^2^⁺, Cd^2^⁺, and Cr^3^⁺, as well as toxic metal oxides like As₂O₃^2^⁺, are common pollutants resulting from human activities. These pollutants arise from illegal wastewater disposal, the use of contaminated fertilizers, manure, sewage sludge, and pesticides in the past, and industrial activities such as mining.^[^ ^500^, ^501^ ^]^ Pollution often involves the coexistence of multiple toxic heavy metal ions, posing a synergistic threat. Additionally, engineered nanomaterials, increasingly used in agriculture, are emerging as a new source of metal contamination. For instance, silver nanoparticles, known for their antimicrobial properties, are being incorporated into packaging materials to prevent spoilage and extend shelf life. They achieve this not only by inhibiting microbial growth but also by breaking down ethylene gas, a compound that accelerates the ripening and spoilage of crops.^[^ ^502^, ^503^ ^]^ Some chemical compounds, such as fulvic acids and humic substances in soil, can coat silver nanoparticles, enhancing their stability and facilitating their transfer to different locations, thereby increasing ecological risks.^[^ ^504^ ^]^ Silver nanoparticles have a strong affinity for sulfur in natural environments, leading to their degradation and the release of Ag⁺ cations in the form of silver sulfides.^[^ ^505^ ^]^

Besides silver nanoparticles, gold nanoparticles and metal oxides such as cerium oxide (CeO₂), titanium dioxide (TiO₂), and zinc oxide (ZnO) are also widely used in agriculture.^[^ ^506^ ^]^ These metals can easily accumulate in the body via the food chain and are harmful to human health even at low concentrations, as they react with sulfhydryl proteins and nucleic acids.^[^ ^507^, ^508^ ^]^ The toxicity of metal cations typically results from their ability to bind tightly to DNA, enzymes, and small organic molecules. The strength of these bonds strongly depends on the chemical nature of the enveloping ligands and the type of metal ion.^[^ ^509^ ^]^ In this section, we will focus on sensors capable of detecting multiple metal ions simultaneously.

**Figure 7:**
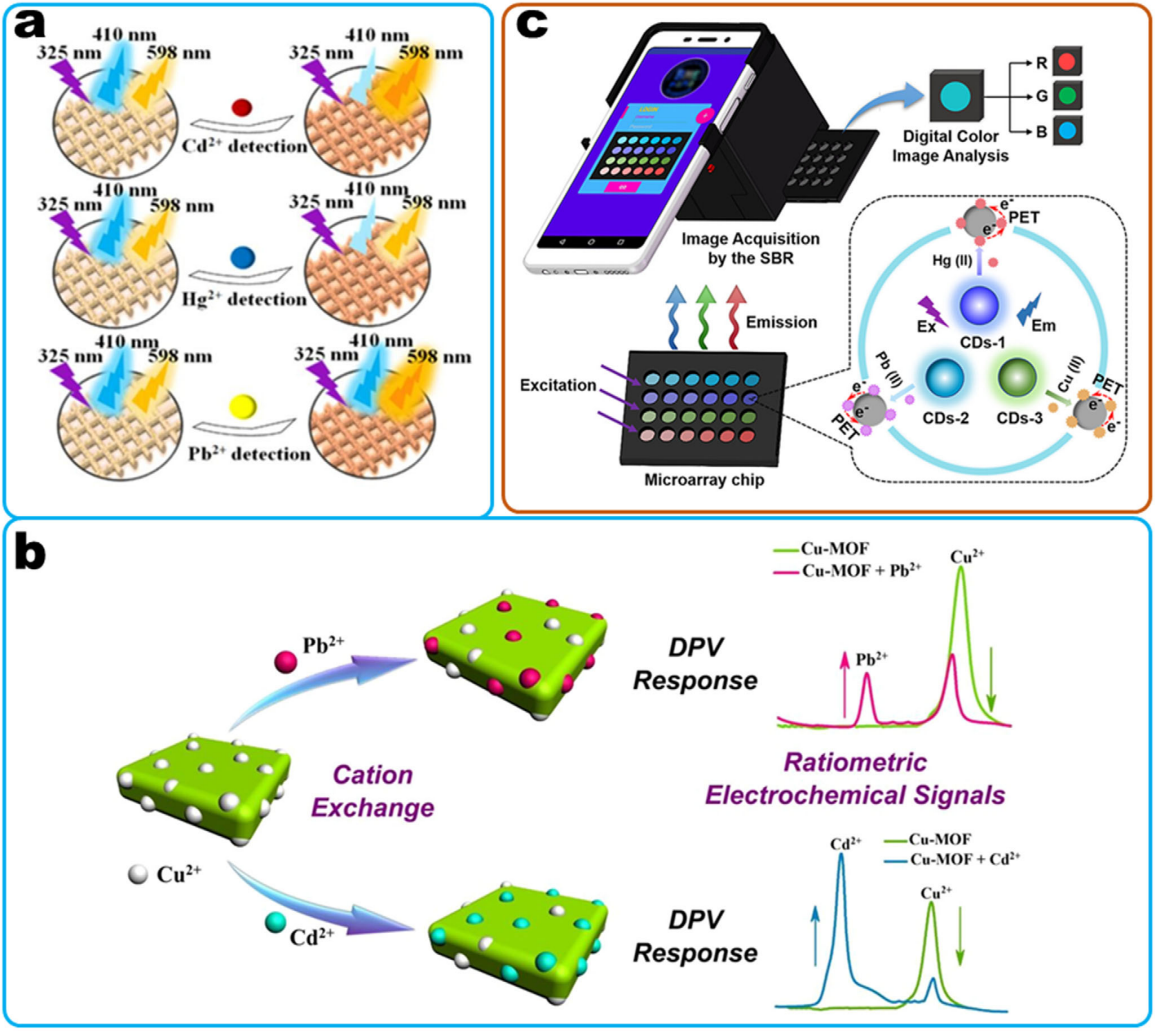
Nanosensors with Multiple Functions for Detecting Metal Ions. a) Paper@MnZnS sensor demonstrating different responses to Cd^2^⁺, Hg^2^⁺, and Pb^2^⁺, utilized for multiplex detection. Reproduced with permission.^[^ ^510^ ^]^ Copyright 2022, Elsevier. b) Schematic illustration of simultaneous detection of Cd^2^⁺ and Pb^2^⁺ by bifunctional MOF materials based on ion‐exchange reaction with differential pulse voltammetry. Reproduced with permission.^[^ ^327^ ^]^ Copyright 2020, American Chemical Society. The nanosensor for multiplex detection was fabricated using nano‐barcode materials. c) Microarray chip fabricated on filter paper using three fluorescent carbon nanodots for detecting Pb^2^⁺, Hg^2^⁺, and Cu^2^⁺, employing a fluorescence “on‐off” model based on the mechanism of non‐radiative electron/hole recombination. Reproduced with permission.^[^ ^516^ ^]^ Copyright 2020, American Chemical Society.

Effective detection and screening of a wide range of metal cations require two critical steps: efficient detection and specific detection, where each sensor unit targets a specific metal ion. For specificity, complex static analyses, such as ML algorithms, are unnecessary. Qin et al. developed a cellulose paper decorated with Mn‐doped ZnS quantum dots (Mn, λ_em_ max = 598 nm) as a sensor for detecting Cd^2^⁺, Hg^2^⁺, and Pb^2^⁺ in water samples (**Figure** **7**a).^[^ ^510^ ^]^ This paper exhibits autofluorescence from lignosulfonate (λ_em_ max = 410 nm), allowing for a ratiometric assay (I_410nm/I_598nm) under excitation, which varies in the presence of different metal ions. The limits of detection for Hg^2^⁺, Pb^2^⁺, and Cd^2^⁺ are 0.01, 0.02, and 1.61 nm, respectively. In this research, Cd^2^⁺ and Hg^2^⁺ interact with lignin, leading to the fluorescent quenching of lignin and the increased fluorescence of QDs due to FRET. In contrast, Pb^2^⁺ interacts only with QDs and does not affect the fluorescence of lignin. Here, a single sensor unit performs multiple functions by using one type of nanoparticle, enabling the identification of these three metal ions by comparing the fluorescent spectra. In essence, this approach integrates multiple single reactions within one sensor.

Some metal oxide nanomaterials exhibit a strong affinity for heavy metal ions due to the crystal plane effect, where unsaturated dangling bonds and surface atom arrangements determine the adsorption and transport of ions.^[^ ^511^ ^]^ For example, α‐Fe₂O₃ nanostructures show strong adsorption with Pb^2^⁺ because the Pb^2^⁺ ions are trapped by three oxygen atoms of α‐Fe₂O₃, enabling detection of Pb^2^⁺ using stripping voltammetry.^[^ ^512^ ^]^ Similarly, Cd and Pb can form intermetallic compounds with Bi and Bi oxide, making Bi₂O₃ a suitable sensor for determining Cd and Pb using electrochemical methods.^[^ ^513^, ^514^ ^]^ Consequently, Fe₂O₃/Bi₂O₃ nanocomposites, which possess multiple reaction abilities, were modified on a glassy carbon electrode to detect Cd^2^⁺ and Pb^2^⁺ simultaneously using stripping voltammetry.^[^ ^515^ ^]^ In another example, Cu^2^⁺ in MOFs such as HKUST‐1 can be replaced by Cd^2^⁺ and Pb^2^⁺ through an ion exchange reaction. Since different metal ions display distinguishable characteristics at different potentials, this MOF was used to detect Cd^2^⁺ and Pb^2^⁺ with differential pulse voltammetry, utilizing the ratio of I_ions/I_Cu^2^⁺ as the signal output (Figure 7b).^[^ ^327^ ^]^

QDs have great potential as barcoded nanomaterials. Yi's research group has developed several point‐of‐care testing (POCT) systems for detecting metal ions.^[^ ^516^, ^517^ ^]^ The chemical groups on CDs can be tuned based on the synthesis precursors, allowing them to bind to different metal ions through specific chelation between the surface molecules and metal ions.^[^ ^518^, ^519^, ^520^ ^]^ Subsequently, the fluorescence of CDs is quenched due to the theory of non‐radiative electron/hole recombination.^[^ ^350^, ^521^, ^522^ ^]^

Xiao et al. synthesized three types of CDs with different emission properties and surface groups: CDs‐1, CDs‐2, and CDs‐3. These were specifically sensitive to Hg^2^⁺, Pb^2^⁺, and Cu^2^⁺ ions, respectively. The method involved the integration of paper‐based microarrays with a portable detector equipped with a smartphone. In the presence of the aforementioned metal cations, the fluorescence of the CDs is quenched. This change in fluorescence can be quantitatively measured using an Android application, which is then used to calculate the concentration of metal cations in water samples from the Pearl River, as shown in Figure 7c.^[^ ^516^ ^]^ By comparing the results with inductively coupled plasma mass spectrometry (ICP‐MS), their methods demonstrated excellent reliability and practicability for on‐site quantitative detection of metal ions.

This strategy of integrating various sensor units on one platform simplifies the detection process. Each nanosensor integrates multiple single specific reactions, enabling both qualitative and quantitative detections without complex data analysis. However, the types of analytes detectable by this method are limited.

The negative impact of toxic metal ions, including both heavy metals and essential metals, on food quality is well known.^[^ ^523^ ^]^ An ideal approach for addressing this issue is the simultaneous detection of as many metal ions as possible. For this purpose, a cross‐reacting strategy proves to be effective. In the context of a sensor array, this strategy involves using different recognition receptors that react to specific ions. These responses are then encoded to create a unique “fingerprint” for each ion.

**Figure 8:**
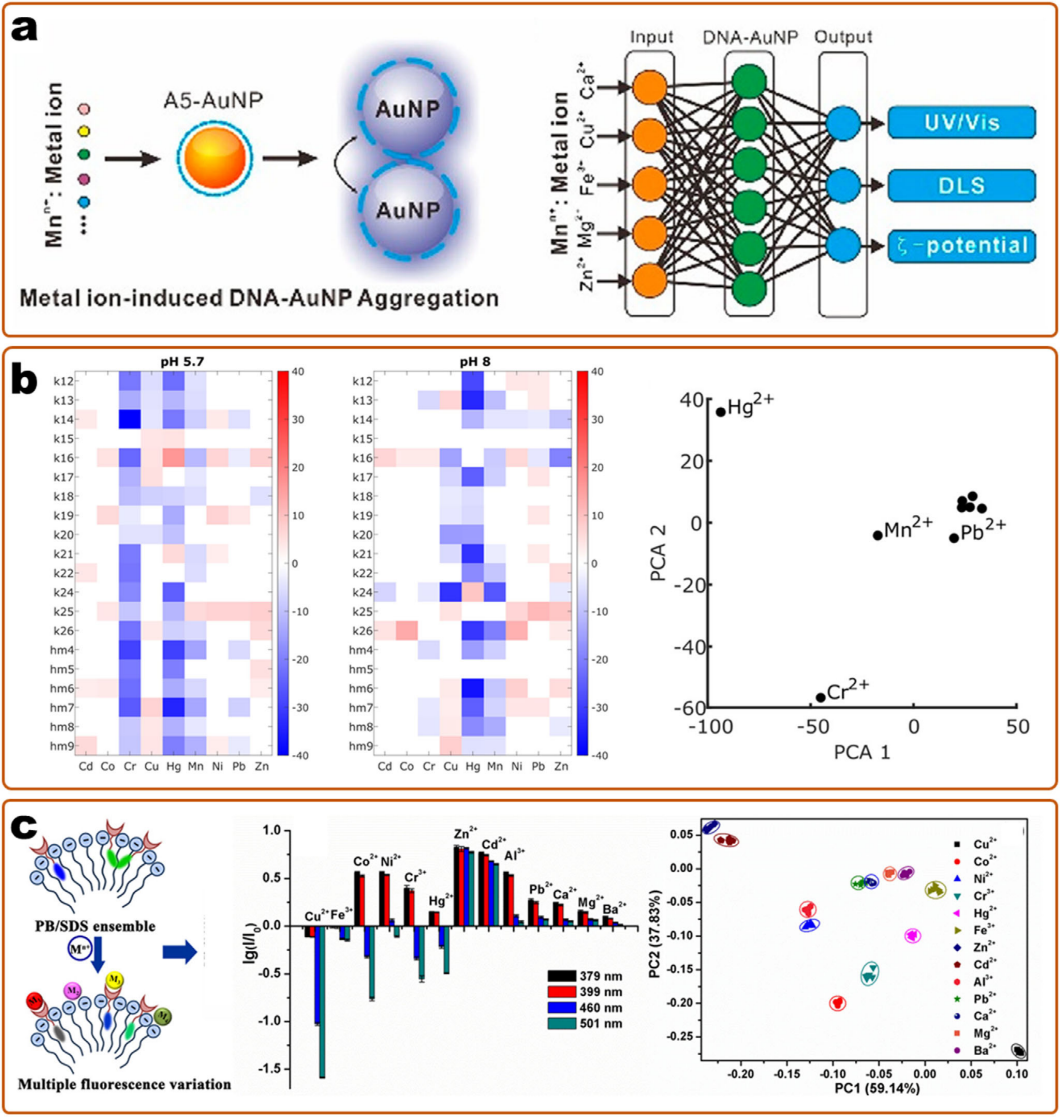
Illustration of Sensor Array with Cross‐Reactivity for Detecting Multiple Ions and Identification by ML Methods. a) Principle of identifying multiple metal ions using DNA‐Au encoders, with A5‐AuNP being one of six types of encoders, analyzed through three output signals: ultraviolet absorption spectrum, dynamic light scattering, and ζ‐potential. Reproduced with permission.^[^ ^524^ ^]^ Copyright 2021, American Chemical Society. b) Sensor responses for different DNA‐SWCNTs to cations under two reaction pH values, allowing discrimination of Hg^2^⁺, Cr^2^⁺, Mn^2^⁺, and Pb^2^⁺ through PCA analysis. Reproduced with permission.^[^ ^525^ ^]^ Copyright 2022, American Chemical Society. c) Schematic illustration of fluorophore/surfactant assemblies‐based cross‐reactive sensor. The four signal recognition patterns for 13 metal ions (at a concentration of 10 µM) were analyzed and discriminated using PCA. Reproduced with permission.^[^ ^527^ ^]^ Copyright 2017, American Chemical Society.

Aizitiaili et al. have developed a remarkable sensor array that consists of gold nanoparticles modified with polyadenines (PolyA) and incorporates machine learning algorithms for high‐throughput detection of multiple metal ions (**Figure** **8**a).^[^ ^524^ ^]^ This method shows great promise for advanced food quality monitoring. PolyA effectively anchors to the surfaces of gold nanoparticles, forming a stable gold colloid. By varying the length of the polyA, the surface charge of the gold nanoparticles can be adjusted, which in turn influences their electrostatic interactions with different metal cations. Specifically, the presence of metal cations such as Ca^2^⁺, Cu^2^⁺, Fe^3^⁺, Mg^2^⁺, and Zn^2^⁺ leads to different aggregation behaviors of the polyA‐modified gold nanoparticles. This aggregation can be observed by optical spectroscopy, as it causes a color change in these plasmonic nanoparticles. It can also be monitored by dynamic light scattering (DLS) and zeta potential measurements. Using linear discriminant analysis, the researchers achieved a detection accuracy of 100% for Ca^2^⁺, Cu^2^⁺, and Mg^2^⁺, and 88% for Fe^3^⁺ and Zn^2^⁺ in concentrations ranging from nanomolar to low micromolar. The method proved effective not only in water, an important medium for food analysis, but also in biofluids such as saliva, urine, cerebrospinal fluid, and blood. This versatility underscores the method's potential for wide‐ranging applications beyond food quality assessment.

Gong et al. used different types of DNA‐coated SWCNTs to develop nanosensors capable of detecting divalent metal cations such as Hg^2^⁺, Pb^2^⁺, Cr^2^⁺, and Mn^2^⁺ in aqueous solutions at nanomolar concentrations, utilizing two different sensor states at pH values of 5.7 and 8 (Figure 8b).^[^ ^525^ ^]^ The sensor response, based on differential fluorescence emission quenching profiles, was analyzed using PCA, enabling the distinction of each metal ion based on different signal patterns. While previous concerns regarding varying chirality distributions in SWCNTs affected calibration curves and signal‐to‐noise ratios, recent advances in SWCNT fabrication have largely mitigated these issues, ensuring a uniform chirality distribution. Importantly, this sensor array has been successfully employed to detect Hg^2^⁺ in fish tissue extracts using a portable detection system.

Ding's research group has made significant advancements in the development of fluorescent cross‐reaction sensors and arrays, particularly by utilizing surfactant assemblies encapsulating fluorophores.^[^ ^526^, ^527^ ^]^ Fluorescently labeled surfactant groups exhibit different fluorescence properties depending on their aggregation state, which is determined by the arrangement of the monomers in a micellar aggregate. Additionally, these surfactant groups maintain a dynamic equilibrium with their monomers and are easily influenced by external stimuli, such as metal ions. Recently, Ding's group reported a single fluorescent sensor based on a newly synthesized pyrene‐modified sodium dodecyl sulfate surfactant, which forms spherical micelles in water.^[^ ^527^ ^]^ The micellar array was used to detect multiple metal ions in drinking water by analyzing the fluorescence signals at four typical wavelengths (501, 460, 399, and 379 nm) (Figure 8c). Their micellar nanosensor demonstrated distinct responses to 13 different metal ions (Ba^2^⁺, Mg^2^⁺, Ca^2^⁺, Pb^2^⁺, Al^3^⁺, Cd^2^⁺, Zn^2^⁺, Fe^3^⁺, Hg^2^⁺, Cr^3^⁺, Ni^2^⁺, Co^2^⁺, and Cu^2^⁺), with the four fluorescence detection patterns effectively distinguished using PCA analysis. The study identified two fluorescence response modes to metal ions. The first mode, termed the “turn‐off” mode, is characterized by fluorescence quenching with increasing metal ion concentration. For example, Cu^2^⁺ reduced excimer emission to 2.26% at 50 µM, and Fe^3^⁺ reduced it to 16.35% at 160 µM, indicating a stronger quenching effect by Cu^2^⁺. The second mode is a ratiometric response, where an increase in monomer emission and a decrease in excimer emission were observed at higher concentrations of certain metal ions, specifically Co^2^⁺, Ni^2^⁺, Cr^3^⁺, Al^3^⁺, and Ca^2^⁺. This dual‐mode detection capability enhances the versatility and sensitivity of the micellar nanosensor for metal ion detection.

A summary of multi‐metal ion analyses is provided in **Table** **4**.

**Table 4:** Summary of nanosensors for detecting multiple metal ions.

Analyte	Sensor system	Detection limit	Environment	Refs.
Cd^2+^, Hg^2+^, Pb^2+^	An optical sensor with cellulose paper modified with Mn‐doped ZnS quantum dots	1.61 nM (Cd^2+^), 0.01 nM (Hg^2+^), and 0.02 nM (Pb^2+^)	Lake water, tap water	[510]
Cd^2+^, Pb^2+^	An electrochemical sensor with carbon paste electrodes modified with Bi_2_O_3_	5 µg/L of both	Drinking water, mineral water and urine	[513]
Pb^2+^, Cd^2+^	An electrochemical sensor based on a hollow sphere bismuth oxide doped mesoporous carbon aerogel nanocomposite	1.72 pM (Pb^2+^), 1.58 pM (Cd^2+^)	Lake water	[514]
Cd^2+^, Pb^2+^	An electrochemical sensor based on Fe_2_O_3_/Bi_2_O_3_ nanocomposites	0.56 nM (Cd^2+^), 0.36 nM (Pb2^+^)	Lake water, tap water, milk	[515]
Hg^2+^, Pb^2+^, Cu^2+^	An optical sensor with carbon nanodots	5.8 nM (Hg^2+^), 0.12 µM (Pb^2+^), 0.076 µM(Cu^2+^)	Spiked water samples, river water	[516]
Cd^2+^, Cu^2+^, Hg^2+^, Pb^2+^	An electrochemical sensor based on screen‐printed carbon electrodes coated with multiple nanomaterials	0.296 µM (Cd^2+^), 0.055 µM (Cu^2+^), 0.351 µM (Hg^2+^), 0.025 µM (Pb^2+^)	Tap water, mineral water, river water	[517]
Ca^2+^, Cu^2+^, Fe^3+^, Mg^2+^, Zn^2+^	A sensor array with multiple DNA‐Au nanoparticles	Working range from 10 nM to 1 mM	Body fluids	[524]
Hg^2+^, Pb^2+^, Cr^2+^, Mn^2+^	A sensor array with single‐walled carbon nanotubes modified by noncovalent corona phases	Working condition, 100 mM	Fish tissue extract	[525]
Cu^2+^, Co^2+^, Ni^2+^, Cr^3+^, Hg^2+^, Fe^3+^, Zn^2+^, Cd^2+^, Al^3+^, Pb^2+^, Ca^2+^, Mg^2+^, Ba^2+^.	A sensor array based on a fluorescent binary ensemble (based on pyrene derivative and sodium dodecyl sulfate assemblies	Working range, 5 µM to 50 µM	Standard solutions	[527]

### 4.2 Chemicals

Molecular contaminants in food mainly include chemical compounds, pesticide residues, industrial chemicals, toxins, hormones, antibiotics, etc., some of which cause side effects and diseases.^[^ ^528^ ^]^ Pesticides are essential to increase agricultural production and meet the needs of the world's growing population.^[^ ^529^ ^]^ The widespread use of pesticides results in irregular misuse and substandard use, which indirectly leads to an imbalance in the ecosystem and the death of beneficial insects and animals. There is a high probability that pesticide residues will end up in processed goods and eventually accumulate in the human food chain. Toxins are harmful to human health, generally including bacterial toxins and mycotoxins. Mycotoxins are widely distributed in food and water and are highly nephrotoxic, carcinogenic, and immunotoxic.^[^ ^530^, ^531^ ^]^ Consequently, the problem of mycotoxin contamination in food is a major concern worldwide, with aflatoxin B_1_ (AFB_1_) and ochratoxin A (OTA) being the two most toxic among the various mycotoxins.^[^ ^532^ ^]^

The extensive production and worldwide utilization of antibiotics are well‐documented, given their critical role in inhibiting bacterial growth. However, the accumulation of antibiotics in the human body, whether originating from human or veterinary medicine, can result in a spectrum of adverse health effects. This pervasive presence of antibiotics underscores the necessity for stringent regulation and monitoring to mitigate potential risks associated with their widespread use.^[^ ^533^ ^]^ In addition, the misuse of antibiotics leads to drug resistance, resulting in less effective treatment of diseases and increasing the risk to human health. After degradation, the metabolism can be more persistent and toxic than that of the parent substance. In general, chemical pollution has often been caused by a specific group of chemicals.^[^ ^534^ ^]^ Humans are continually exposed to a complex mixture of chemicals, only a few of which have been categorized. With the introduction of the exposome concept, the requirements for handling complex mixtures of chemicals have been extended. Notably, mixtures of chemicals can exert toxic effects if they are below the analytical detection limit or their own effect threshold.^[^ ^535^ ^]^ This phenomenon underscores the growing necessity for comprehensive monitoring of multichemical residues. Moreover, in the context of food authenticity and traceability, there is a pressing need for efficient methods to identify components within complex mixtures. These methods must be cost‐effective and capable of discerning subtle distinctions among adulterated foods.

**Figure 9:**
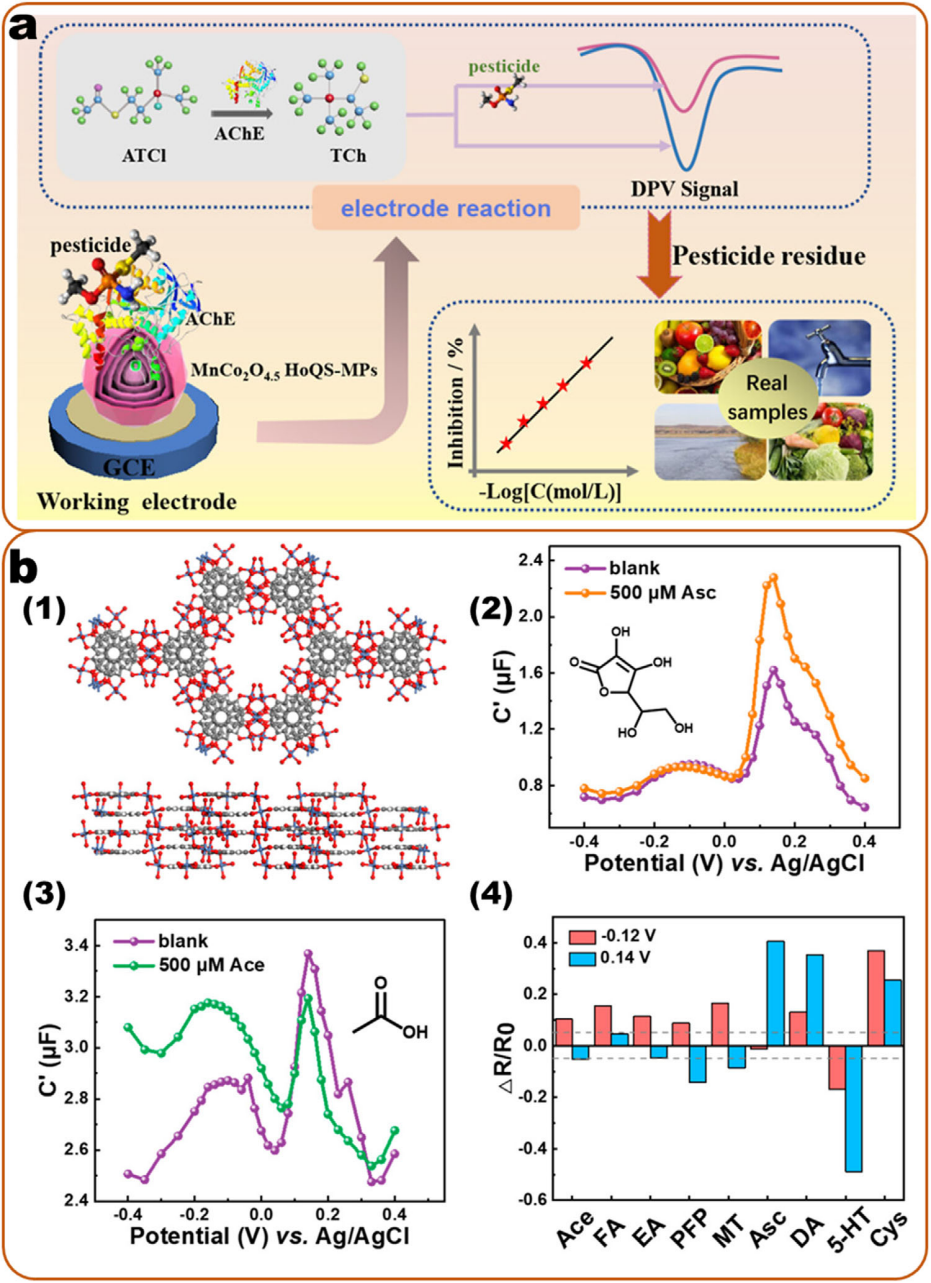
Representative examples of multiplex detection of chemicals using a single nanosensor. a) Illustration of multipesticide residue detection (monocrotophos, methamidophos, and carbaryl) based on the inhibition of acetylcholinesterase activity, utilizing an electrochemical method. Reproduced with permission.^[^ ^536^ ^]^ Copyright 2023, American Chemical Society. b) Detection principle for small molecules using Ni‐HHTP as the sensing unit, which exhibits two capacitance peaks: (1) structure of Ni‐HHTP, (2‐3) capacitance of Ni‐HHTP, and Ni‐HHTP in the presence of ascorbic acid (Asc) and acetic acid (Ace). c) Relative capacitance response in the presence of different analytes, including ascorbic acid (Asc), acetic acid (Ace), formic acid (FA), ethylamine (EA), perfluoropropionic acid (PFP), melatonin (MT), dopamine (DA), and cysteine (Cys). Reproduced with permission.^[^ ^538^ ^]^ Copyright 2023, American Chemical Society.

Certain chemicals share structural similarities that can be identified through specific reactions. For instance, organophosphate and carbamate pesticides both react with serine in acetylcholinesterase via phosphorylation. Based on this phenomenon, multiplex detection can be realized in one sensor. Song et al. developed 2D conductive transition metal oxides (TMOs) with a hollow multi‐shell structure, exhibiting diverse electronic and electrical properties as electrodes. They selected acetylcholinesterase (AChE) as the recognition element. In the presence of methamidophos, monocrotophos, and carbaryl, the activity of AChE is inhibited, leading to a decrease in the electrical signal (**Figure** **9**a).^[^ ^536^ ^]^ This sensor has also been effectively employed to detect pesticides in pakchoi samples spiked with concentrations of 0.5, 5, and 10 nM, achieving promising recovery rates ranging from 90.0% to 115.6%. Similarly, the phosphate ester of organophosphorus pesticides (OPs) can interact with an aptamer possessing a neck ring structure to form a G‐triplex. This interaction facilitates the detection of OPs through an electrochemical method.^[^ ^537^ ^]^

These studies have demonstrated the ability to recognize particular classes of chemicals, yet they fall short of identifying the specific structures of these chemicals, highlighting a gap in our understanding of multiplex detection. Building on a similar design strategy, Guo et al. utilized 2D conductive metal–organic frameworks (Ni‐HHTP) for electrochemical sensing. These frameworks were synthesized using Ni(II) as metal nodes and 2,3,6,7,10,11‐hexahydroxytriphenylene (HHTP) as organic ligands (Figure 9b).^[^ ^538^ ^]^ At very low voltages, this material displays two capacitive peaks, which can be employed as dual detection channels.

Intriguingly, various redox‐active and redox‐innocent compounds interact with Ni‐HHTP, inducing different redox states and causing fluctuations in capacitance, thereby forming distinct signal patterns. This approach surpasses previous examples by achieving the identification of different chemicals.

**Figure 10:**
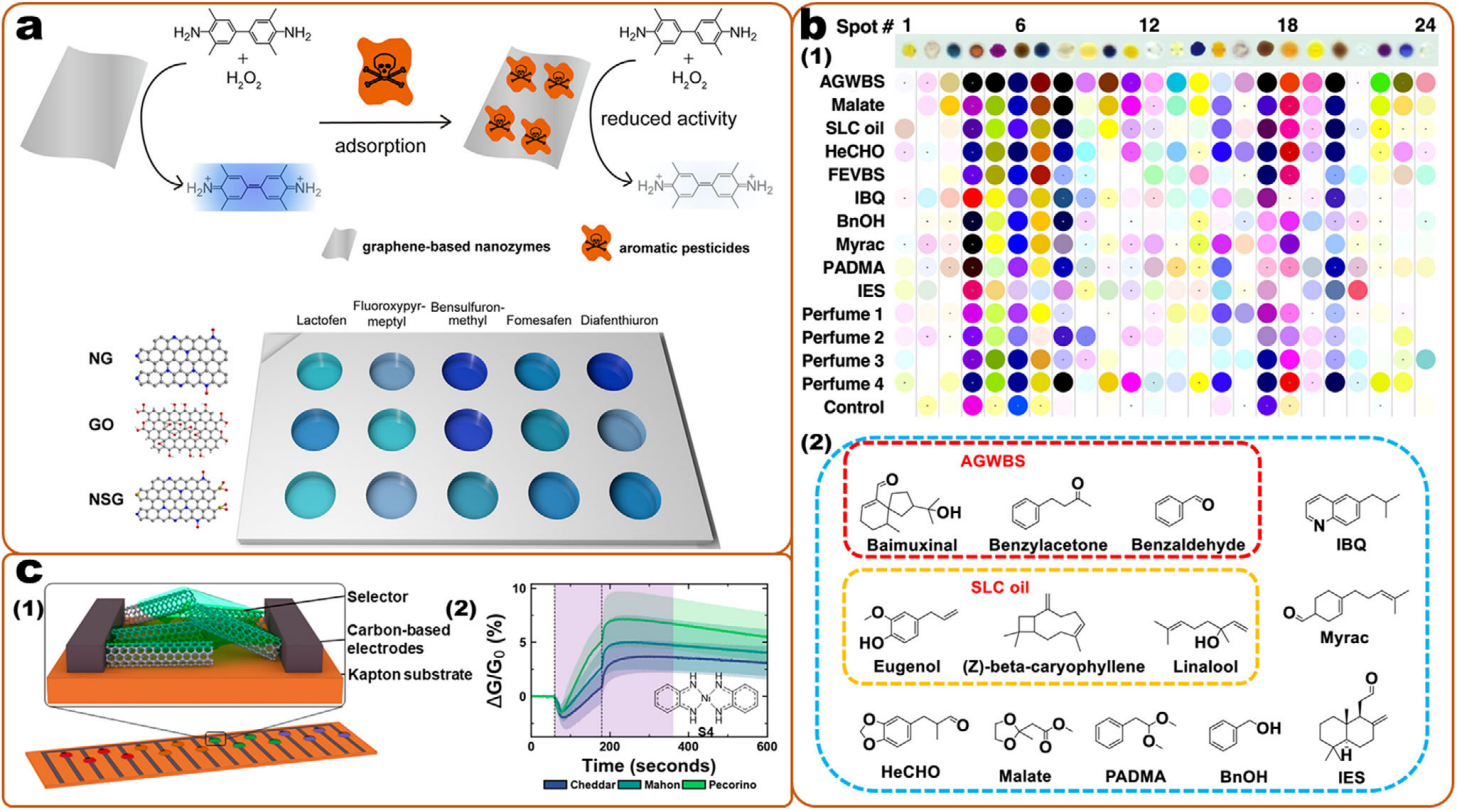
Illustration of detecting multiple chemicals using sensor arrays. a) Principles of nanoenzyme sensor arrays for detecting aromatic pesticides based on heteroatom‐doped graphene. The catalytic activity of the nanoenzyme is inhibited to varying degrees by different pesticides, resulting in distinct colors of catalytic reactions. Reproduced with permission.^[^ ^539^ ^]^ Copyright 2020, American Chemical Society. b) A sensor array containing 24 sensing units for discriminating perfumes: (1) sensor responses to perfume samples, (2) the formulas of typical aromatic components in the perfume bases. Reproduced with permission.^[^ ^540^ ^]^ Copyright 2022, American Chemical Society. c) Schematic of classifying food with a chemiresistive sensor array based on multi‐electrodes modified by SWCNTs and selectors as the active layer: (1) composition of the sensing device, including carbon‐based electrodes and polyimide substrate, (2) examples of one selector interacting with cheddar, Mahon, and pecorino, with conductance as the output signal. Reproduced with permission.^[^ ^159^ ^]^ Copyright 2019, American Chemical Society.

Sensor arrays discriminate various analytes for multiplex detection by generating specific signal patterns. Zhu et al. reported a nanozyme sensor array based on heteroatom‐doped graphenes for detecting pesticides with aromatic structures (**Figure** **10**a).^[^ ^539^ ^]^ This work features a nanozyme‐like sensor array composed of three types of graphene materials: nitrogen‐doped graphene (NG), nitrogen‐ and sulfur‐codoped graphene (NSG), and graphene oxide (GO). These materials exhibit peroxidase‐like activities, enabling a catalytic reaction with 3,3′,5,5′‐tetramethylbenzidine dihydrochloride (TMB), and H_2_O_2_, producing a blue color change. In the presence of aromatic pesticides, the colorimetric reaction is altered as these pesticides adsorb to the active sites of the nanoenzymes, reducing their peroxidase‐like activity. The affinity of each pesticide to the sensing units varies, resulting in unique colorimetric response patterns. These patterns were analyzed and converted into canonical 2D score plots using LDA, producing distinct clusters for each of the five pesticides. This methodology demonstrated the sensor array's ability to accurately identify pesticides in real soil samples across a concentration range of 10–1000 µM. The successful discrimination of pesticides, even amidst other substances and under real sample conditions, underscores the potential of these nanozyme sensor arrays for practical applications.

For samples containing a large number of components, a sensor array with a greater number of detection units is typically required. Perfumes, for instance, often comprise 100–2000 organic components. A remarkable example of a nanosensor capable of handling such complexity was reported by Li's group.^[^ ^540^ ^]^ In this study, a colorimetric sensor array with 24 different optically responsive inks was employed for the detection and discrimination of common fragrance precursors and real perfumes (Figure 10b). The inks used were composed of previously reported solvatochromic and reactive probes based on intermolecular interactions.^[^ ^202^, ^541^ ^]^ The design of the sensor array features a streamlined gas flow channel, ensuring rapid gas exposure across all elements, which is completed within 15 s. The visual color changes of the sensor array were digitally recorded, capturing the response of the patterned array. These responses were then analyzed using standard chemometric techniques such as HCA and PCA. This approach allowed for the effective identification and differentiation of complex fragrance compositions, demonstrating the sensor array's robustness and potential for practical applications in detecting and analyzing intricate chemical mixtures. The array successfully distinguished four commercial perfume samples by categorizing them into different clusters based on brand and ingredient similarities with their respective fragrance bases. Furthermore, in proof‐of‐concept experiments, the sensor array proved capable of identifying unadulterated perfumes from samples diluted with different amounts of ethanol. Generally, in these sensor arrays with many sensing units, only the chemical binding elements can meet the requirement of fabricating a large number of sensing channels. For instance, transition metal complexes can interact with organic acids;^[^ ^542^ ^]^ sulfur‐containing compounds and ionic liquids can bind aromatic compounds, alkanes, aldehydes, and ketones;^[^ ^543^ ^]^ metalloporphyrins can react with aromatic compounds, alkanes, ketones, alcohols, and amines;^[^ ^544^, ^545^ ^]^ cavitand molecules, with their size exclusion properties, can detect alcohols and aromatic compounds;^[^ ^546^, ^547^ ^]^ and porous polymers can absorb organic vapors.^[^ ^548^, ^549^ ^]^ These diverse chemical interactions enable the sensor arrays to effectively handle the complexity of multi‐component samples, enhancing their applicability in real‐world scenarios.

Based on these chemical reactions, Schroeder et al. reported a 20‐element sensor array constructed with SWCNTs to differentiate food samples, including cheese, edible oil, and liquor, based on their odor (Figure 10c).^[^ ^159^ ^]^ In this study, a novel approach was developed using 20 carbon nanotube‐based chemical sensors in a sensor array to detect complex odors, specifically distinguishing between cheese, liquor, and cooking oil. The method involved a two‐stage ML process. First, an optimal sensor subset was identified for each category, followed by validation on an extended dataset. The optimal selectors were chosen based on independent classification accuracy and analysis of 4845 possible selector combinations. The classification was performed using K‐nearest neighbor and random forest models, resulting in high accuracy rates for independent test sets: 91% for cheese, 78% for spirits, and 73% for edible oil. This research provides a generalizable method with potential applications in various fields, including disease diagnostics, hazard detection, and food authentication. Moreover, it lays the groundwork for future research in effective selector identification for complex gas sensing problems.

**Figure 11:**
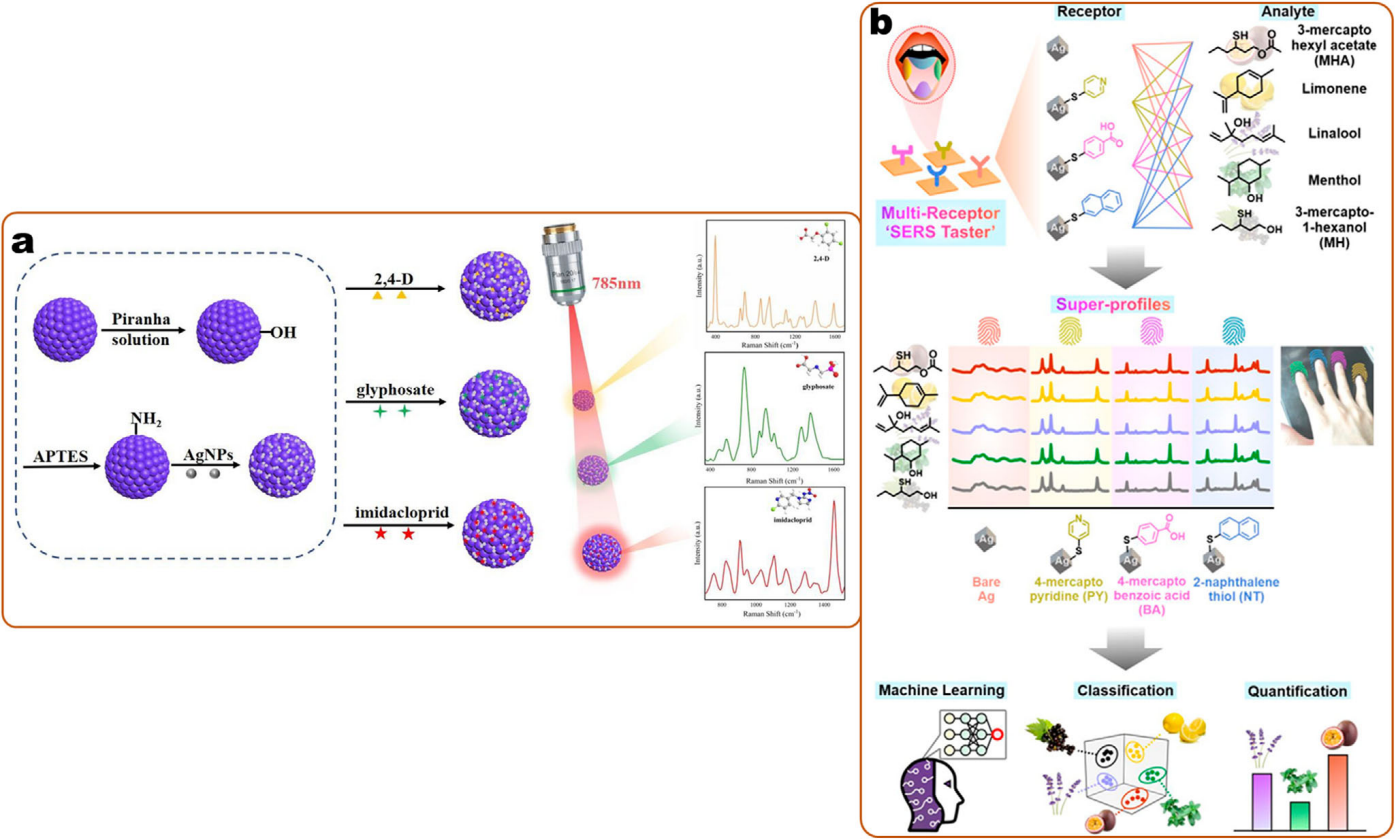
Examples of multiplex detection of chemicals using label‐free SERS methods. a) Simultaneous detection of multiple pesticides (2,4‐dichlorophenoxyacetic acid (2,4‐D), glyphosate, and imidacloprid) utilizing a 3D Ag‐silica photonic microsphere array. Reproduced with permission.^[^ ^552^ ^]^ Copyright 2023, American Chemical Society. b) Principles of a SERS array for identifying and quantifying wine flavor molecules with the assistance of ML. This sensor array comprises four sensing units, each capable of binding target molecules to form a SERS superprofile. Data analysis is performed using ML chemometric models for precise identification and quantification. Reproduced with permission.^[^ ^104^ ^]^ Copyright 2021, American Chemical Society.

SERS spectra provide fingerprint information of molecules, offering highly concrete and precise data. Label‐free SERS methods are widely employed for identifying chemical structures, ranging from individual molecules to whole cells.^[^ ^260^, ^550^ ^]^ These techniques are particularly well‐suited for chemical identification and detection, making them invaluable tools in various analytical applications.^[^ ^551^ ^]^ Xu et al. prepared a silver‐coated silica microsphere array as a SERS‐enhanced substrate.^[^ ^552^ ^]^ This setup enabled the detection of pesticides such as 2,4‐D, imidacloprid, and glyphosate, which generated distinct characteristic peaks when they entered the “hot spots” (**Figure** **11**a). Similarly, Li et al. reported a 3D‐nanocauliflower SERS substrate for the simultaneous label‐free detection of aflatoxin B1, zearalenone, and deoxynivalenol.^[^ ^553^ ^]^ The characteristic peaks facilitated both quantitative and qualitative analysis. However, complex targets like tea differentiation involve a vast array of chemicals, leading to overlapping and chaotic characteristic peaks. In such cases, the accuracy of analyte discrimination can be significantly improved by increasing the dimensionality of the spectral data.^[^ ^554^ ^]^

In this context, ML can significantly enhance the classification of variations and achieve high predictive accuracies. However, there is a risk of model overfitting, which can result in failures when predicting unknown samples. To mitigate this risk, it is crucial to understand the spectral variations stemming from chemical interactions and to establish a robust correlation between ML and chemical knowledge, thereby improving predictive accuracy. Leong et al. addressed this by selecting four receptors to capture typical flavor molecules through noncovalent interactions.^[^ ^104^ ^]^ The spectra from all receptor‐flavor interactions were compiled into a “SERS superprofile.” PCA and SVM discriminant analysis were then employed to analyze this superprofile and construct models, enabling multiplex quantification and identification of wine flavors with high accuracy (Figure 11b).

A summary of multi‐chemical analyses is provided in **Table** **5**.

**Table 5:** Summary of nanosensors for detecting multiple chemicals.

Analyte	Sensor system	Detection limit	Environment	Refs.
Monocrotophos, methamidophos, carbaryl	An electrochemical sensor based on MnCo_2_O_4.5_ hollow quadruple‐shelled porous micropolyhedron	1.82 × 10^−14^ M (MMonocrotophos) 1.66 × 10^−14^ M (methamidophos) 1.58 × 10^−14^ M (carbaryl)	Pakchoi	[536]
Organophosphorus pesticides	An optical sensor based on Fe‐Co nanoporous	7.32 fM	Vegetables	[537]
Formic acid, ethylamine, perfluoropropionic acid, melatonin, ascorbic acid, acetic acid	An electrochemical sensor based on 2D conductive metal–organic frameworks	The limit of detection is different as each target	Standard solutions	[538]
Lactofen, fluoroxypyr‐meptyl, bensulfuron‐methyl, fomesafen, and diafenthiuron	A sensor array with nanoenzymes	Working range, 0–1 mM	Soil samples	[539]
Fragrance bases and perfume products	A sensor array based on multiple dys	Qualitative identification	Perfumes	[540]
Human breath‐related volatile organic compounds	A sensor array based on carbon nanotube	Qualitative identification	Health diagnosis	[543]
Volatile organic compounds	A sensor based on multiwalled carbon nanotube	Qualitative identification	In lab environment	[546]
Menthol, linalool, limonene, 3‐mercaptohexyl acetate, 3mercapto‐1‐hexanol	A sensor away based on Au nanoparticles	Qualitative identification	Wine	[104]
Volatile organic compounds	A sensor array based on single‐walled carbon nanotube	Qualitative identification	Edible oil	[159]
Saccharin sodium, carmine acid	A sensor based on GO‐coated Fe_3_O_4_@TiO_2_@AuAg SERS substrate	2.1 µg/mL (saccharin sodium), 99.7 nM (carmine acid)	Preserved fruit, hawthorn, soda and juice	[551]
2,4‐dichlorophenoxyacetic acid, glyphosate, imidacloprid	A sensor based on Ag@three‐dimensional silica photonic microsphere array	3.03 ng/mL (2,4‐D), 3.14 ng/mL(glyphosate), 8.82 ng/ mL(imidacloprid)	Tap water, soybean, apple, tomato	[552]
Aflatoxin B1, zearalenone, deoxynivalenol	A sensor based on 3D SERS substrate	1.8 ng/mL (aflatoxin B1), 47.7 ng/mL (zearalenone), 24.8 ng/mL (deoxynivalenol)	Corn	[553]

### 4.3 Foodborne Pathogens

Biological contaminants, including parasites, viruses, bacteria, and prions, are the most common causes of foodborne illnesses. According to the WHO, pathogens such as *Campylobacter, Salmonella*, and *Escherichia coli* impact millions of individuals annually. These pathogens are commonly found in eggs, milk, drinking water, fresh fruits, and vegetables. Additionally, *Listeria* and *Vibrio cholerae* are prevalent in contaminated food, posing serious health risks. Notably, *Listeria* can proliferate even at refrigerator temperatures, further complicating efforts to ensure food safety.^[^ ^555^ ^]^ Antibiotics are necessary to treat infections caused by bacteria, but increasing bacterial resistance is a major challenge associated with the misuse of antibiotics.^[^ ^533^, ^556^ ^]^ Food‐ and waterborne viruses also contribute to 21% of all foodborne diseases and 8.3% of deaths related to foodborne illnesses.^[^ ^557^ ^]^ Bacteria are single cells that can survive on their own, while viruses are much smaller and need a host to replicate. According to the type of illness, foodborne viruses are divided into three groups: fecal‐orally transmitted hepatitis viruses (hepatitis E and hepatitis A virus), viruses causing gastroenteritis (Norwalk‐like viruses, rotavirus, norovirus, typical caliciviruses, and enteric adenovirus), and viruses surviving in the intestine. These viruses, along with bacterial pathogens, significantly contribute to the global burden of foodborne diseases.^[^ ^557^, ^558^, ^559^ ^]^ In addition to the most common viruses, the development of food trade and cold chain transportation, coupled with the global epidemic of SARS and COVID‐19, has highlighted other viruses, such as COVID‐19, as significant threats to food safety and human health.^[^ ^560^ ^]^ Bacteria, with sizes reaching micrometers, can be detected using various methods. Among these, immunoassays are particularly advantageous due to their low cost and ease of use.^[^ ^561^ ^]^ Viruses consist of an outer protein coating (capsid) and a nucleic acid core (double‐ or single‐stranded DNA or RNA). The analytes for detection can be the virus itself or the antibodies, proteins, nucleic acids, and enzymes produced by the virus.

Widely accepted methods for identifying microorganisms rely on molecular biotechnology, such as PCR, and culture‐based methods, which require sophisticated instruments or long processing times. In the detection process using nanosensors, bioreceptors—including nucleic acids, antibodies, aptamers, proteins, or peptides—are commonly employed. These bioreceptors interact with analytes, causing chemical or physical changes that generate quantifiable optical or electrical signals. However, achieving highly selective receptors for each microorganism or biomolecule in complex matrices is challenging due to cross‐reactivity, leading to common occurrences of false positive results. This remains a significant hurdle in the field of nanosensor‐based detection.^[^ ^562^, ^563^ ^]^ In addition, traditional “one‐to‐one” recognition approaches are inefficient for discriminating subtle differences in subtypes. Despite this limitation, the antibody‐antigen reaction remains one of the most popular methods due to its established techniques.^[^ ^8^, ^561^, ^564^, ^565^ ^]^

**Figure 12:**
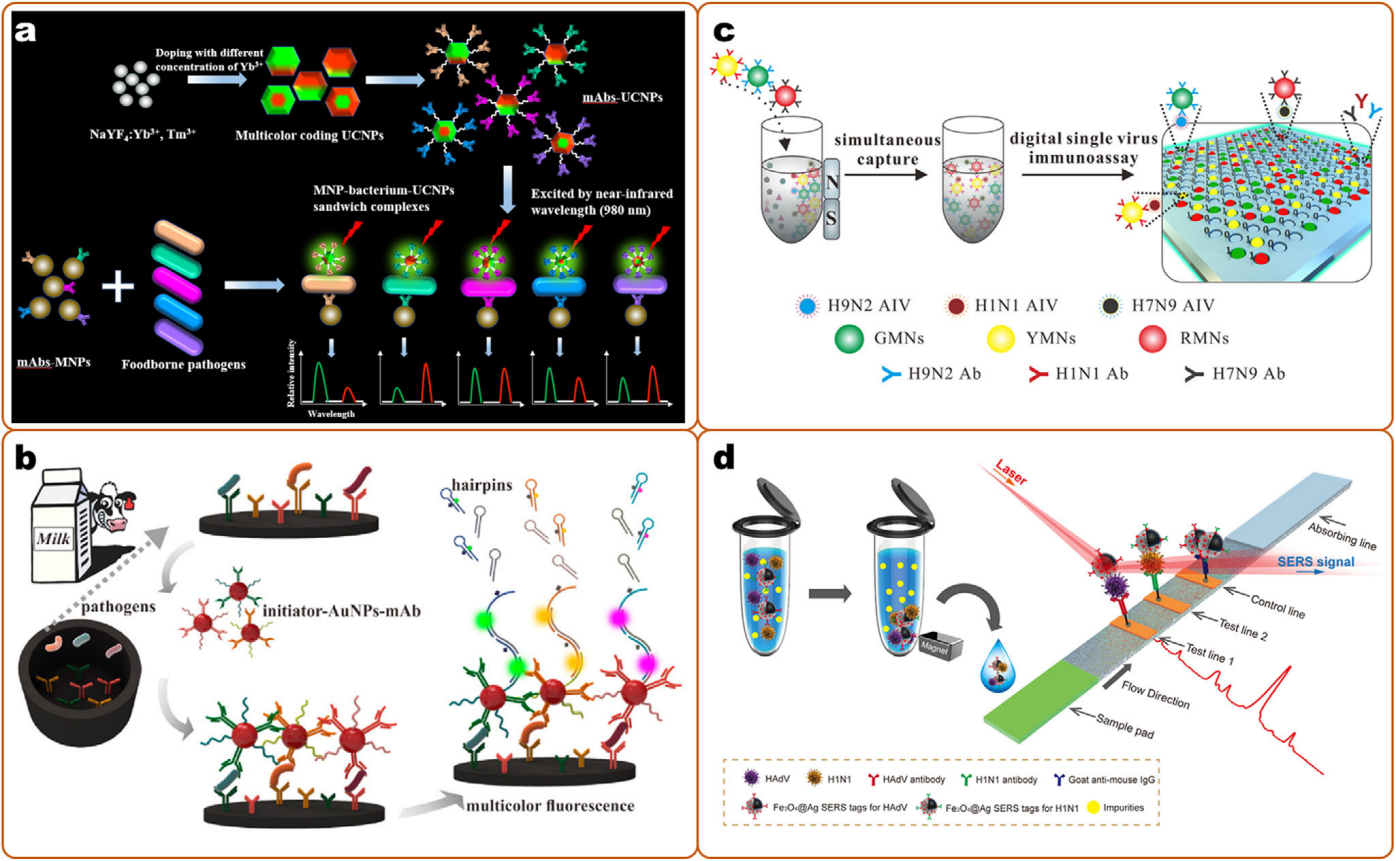
Illustrative Examples of Nanosensors for Pathogen Detection. The figure showcases various nanosensors used for pathogen detection, incorporating multiple specific single detection reactions. a) Multicolor Coding UCNPs: Functionalized with antibodies for detecting five types of pathogens based on immunoassay. Reproduced with permission.^[^ ^568^ ^]^ Copyright 2021, American Chemical Society. b) Ultrasensitive Enzyme‐Linked Immunosorbent Assay (ELISA): Utilizes HCR and multicolor fluorescent dyes for the detection of three pathogenic bacteria. Reproduced with permission.^[^ ^572^ ^]^ Copyright 2019, American Chemical Society. c) Single Virus Immunoassay: Employs multifunctional QD‐decorated magnetic nanospheres for the detection of three avian influenza viruses. Reproduced with permission.^[^ ^575^ ^]^ Copyright 2019, American Chemical Society. d) Lateral Flow Immunoassay: Detects two respiratory viruses using two antibody‐modified Fe₃O₄@Ag magnetic tags. Reproduced with permission.^[^ ^576^ ^]^ Copyright 2019, American Chemical Society.

With the advancements in nanotechnology, biosensing strategies have been enhanced by various types of nanosensors. Multiplex analyses are particularly effective for detecting different bacteria, which, although similar in composition, have different subtypes. However, due to the size of bacteria and viruses, it is challenging to detect multiple targets simultaneously with a single sensor. Integrating multiple binding units into a single sensor is generally not feasible for the detection of multiple microorganisms. Instead, approaches such as using barcoded nanomaterials, multiple detection positions, and sensor arrays are more common. For instance, the luminescence of up‐conversion nanoparticles (UCNPs) can be adjusted by altering their composition.^[^ ^566^ ^]^ For example, by adjusting the Yb^3^⁺ doping concentration, UCNPs with varying red/green luminescence ratios can be produced.^[^ ^567^ ^]^ Hu et al. developed a multicolor coding up‐conversion nanoplatform for the detection of *Salmonella choleraesuis*, *S. paratyphic C, Salmonella enteritidis, Salmonella paratyphi B and E. coli O157:H7*. Five types of monoclonal antibodies (mAbs) against these foodborne bacteria were conjugated to five distinct UCNPs. The detection process involved capturing the target bacteria with mAb‐modified magnetic nanoparticles, followed by the addition of the mAb‐modified UCNPs to form a sandwich structure. By measuring the red/green ratio and green photoluminescence (PL) intensity, the bacteria could be distinguished and quantitatively detected (**Figure** **12**a).^[^ ^568^ ^]^ The DNA hybridization chain reaction (HCR) is an efficient and straightforward isothermal process for multiplex signal amplification. It involves the use of initiator DNA and at least two hairpin‐structured DNA molecules (H1 and H2).^[^ ^569^, ^570^, ^571^ ^]^ Lv et al. utilized fluorescent dyes as coding elements for the simultaneous detection of pathogens based on an immunoassay combined with DNA HCR. In this approach, gold nanoparticles (AuNPs) labeled with mAbs and initiator DNA were fabricated as probes for *Listeria monocytogenes, Salmonella serotype Choleraesuis, and Escherichia coli O157:H7*. Fluorophore and quencher‐modified hairpin structures (H1) were employed in the HCR process. Upon interaction with the target bacteria, the hybridization chain reaction was triggered, enabling sensitive and multiplex detection. (Figure 12b).^[^ ^572^ ^]^ QDs possess strong fluorescence intensity and stability compared to traditional fluorescent dyes, with size‐ and composition‐dependent optical properties that make them superior for optical encoding.^[^ ^573^, ^574^ ^]^ Wu et al. reported a fluorescent method based on magnetic nanoparticles covered by QDs.^[^ ^575^ ^]^ By modifying these nanoparticles with antibodies for H1N1, H7N9, and H9N2 avian influenza viruses, they developed three fluorescent magnetic multifunctional nanospheres (yellow, red, and green) for virus detection, serving as both signal labels and capture carriers. Due to their excellent fluorescent imaging and photostability, single‐particle counting for multiplex detection was achieved (Figure 12c). This design offers strong anti‐interference ability, high sensitivity, good specificity, and universality for multiplex detection. By amplifying the number of encoded nanospheres, this approach can detect more types of targets simultaneously. Lateral flow immunoassay strips are popular POCT techniques due to their easy production, simple operation, low cost, and short assay time. It is possible to add more test lines to the strip for enhanced functionality. Wang et al. used Fe₃O₄@Ag nanoparticles as substrates, modifying them with various SERS tags and virus antibodies separately to create capture units. These units were used to enrich the analytes (H1N1 and HAdv) (Figure 12d).^[^ ^576^ ^]^ The nanoparticles‐virus composites were trapped on the test lines, and detection was realized through the characteristic SERS peak of Fe₃O₄@Ag tags. Given that viruses are much smaller, achieving single‐molecule detection (SMD) would allow for detailed exploration of their physicochemical characteristics. However, an approach with high spatial resolution and sensitivity relies on collecting and amplifying weak signals from single molecules. Compared to other methods, the application of lateral flow immunoassay offers much more convenient multiplex detection. The innovative design of fluorescent magnetic multifunctional nanospheres enables ultrasensitive detection.

In practice, an affordable and straightforward approach is more attractive, especially in resource‐limited areas. However, all these design strategies fundamentally involve collecting multiple specific single‐detection reactions, adhering to the traditional “lock and key” principle. To achieve multiplex detection of pathogens, it is essential to have sufficient binding elements with high specificity. Although the potential number of nano‐barcodes is vast, developing enough highly selective binding elements, such as antibodies and aptamers, remains a significant challenge.

**Figure 13:**
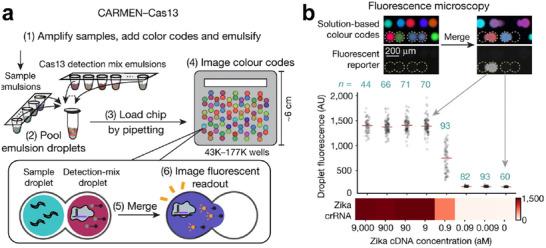
Illustrative Examples of the CARMEN‐Cas Workflow for Virus Detection. a) CARMEN‐Cas Workflow: Illustration of the workflow capable of detecting 169 viruses simultaneously. b) Zika cDNA Detection: Example of sensitively detecting Zika cDNA using a single CARMEN‐Cas13 assay. Reproduced with permission.^[^ ^590^ ^]^ Copyright 2020, Springer Nature.

Sensitivity and selectivity are the primary objectives of analytical chemistry. In this context, the CRISPR‐Cas system represents a significant advancement, especially given its potential for single‐molecule detection and point‐of‐care applications. CRISPR stands for Clustered Regularly Interspaced Short Palindromic Repeats. These repetitive DNA sequences were first discovered in bacteria, where they precisely match viral sequences. Upon viral infection, these DNA elements are transcribed into RNA, which then guides a nuclease to cut the viral DNA, thereby providing protection against the infection. These nucleases are known as ‘Cas‘ proteins.^[^ ^577^, ^578^, ^579^, ^580^ ^]^ The CRISPR/Cas system can be activated by foreign genetic elements with excellent selectivity.^[^ ^581^ ^]^ The Cas proteins, such as Cas9, Cas13, Cas12, and Cas14, are widely used in gene editing and have significantly advanced the potential of POCT.^[^ ^582^ ^]^ Cas9 can specifically cut targeted double‐stranded DNA, while Cas12 and Cas13, once activated by their target, can also cleave surrounding single‐stranded DNA or RNA.^[^ ^583^, ^584^, ^585^, ^586^ ^]^ Due to its simple design, high efficiency, low cost, and exceptional selectivity, the sensing method based on the CRISPR/Cas system is rapidly gaining prominence. This system is revolutionizing analytical chemistry, enabling highly sensitive and selective detection that is critical for various applications, from advanced research to practical diagnostics.^[^ ^587^, ^588^, ^589^ ^]^ M.Ackerman et al reported a combinational arrayed reactions for multiplex evaluation of nucleic acids (CARMEN) platform, capable of simultaneously recognizing all 169 human‐associated foodborne viruses (**Figure** **13**).^[^ ^590^ ^]^ In this research, an RNA probe modified with a fluorophore and quencher at the ends was designed. In the presence of targeted RNA, Cas13 activated and initiated fluorescence. Combined with specific high‐sensitivity enzymatic reporter unlocking (SHERLOCK), the target RNA was amplified. Subsequently, the detection mixture (Cas13, guide RNA, and fluorescent RNA probe) was added, activating the fluorescence. The target RNA was identified based on the fluorescence signal. In the CARMEN platform, each sample was labeled with a fluorescent dye (green or blue) and packaged in emulsions. Each detection mixture was similarly labeled with a red or orange dye. Sample droplets and detection droplets were then mixed and rolled out in a specialized well plate, allowing each sample droplet to mix with a detection droplet. The detection process was monitored using a fluorescent microscope. This color‐coded platform could test more than 4,500 CRISPR RNA pairs on a single array. Compared to RT‐PCR and high‐throughput sequencing, this technique demonstrated higher sensitivity and reliability.

It is very complicated to create highly selective binding elements for each bacterium in a sample containing a complex mixture of bacteria. The traditional “lock and key” strategies are often inadequate for distinguishing subtle differences within such complex bacterial mixtures.^[^ ^591^ ^]^ In addition, pathogenic microorganisms have many subtypes, and the speed of developing new biorecognition elements lags behind the discovery of new pathogens. For example, pathogenic *E. coli* can be divided into six categories, each containing various pathotypes.^[^ ^592^ ^]^

It is challenging to implement pre‐prepared detection methods capable of identifying potential hazards comprehensively. The sensor array strategy offers a promising solution by expanding detection capacity, enabling the identification of a broader range of threats simultaneously.^[^ ^462^, ^593^ ^]^ Bacteria differ in their metabolites and surface physicochemical properties, such as the cis‐diol group of saccharides, lectin proteins, liposomes, and D‐Ala‐D‐Ala dipeptides, which can be utilized for identification. Additionally, noncovalent or specific interactions between bacteria and nanomaterials, such as hydrophilic/hydrophobic and electrostatic interactions, also facilitate bacterial differentiation.^[^ ^594^ ^]^ Here, the non‐specific binding elements generally include boronic acid, glycan, peptides, glycopeptide antibiotics, and some proteins.^[^ ^595^, ^596^ ^]^ By combining versatile recognition principles, various signal patterns can be produced to identify bacteria. The bacterial surface is rich in negative charges, making it a good competitor for binding with positively charged materials.

**Figure 14:**
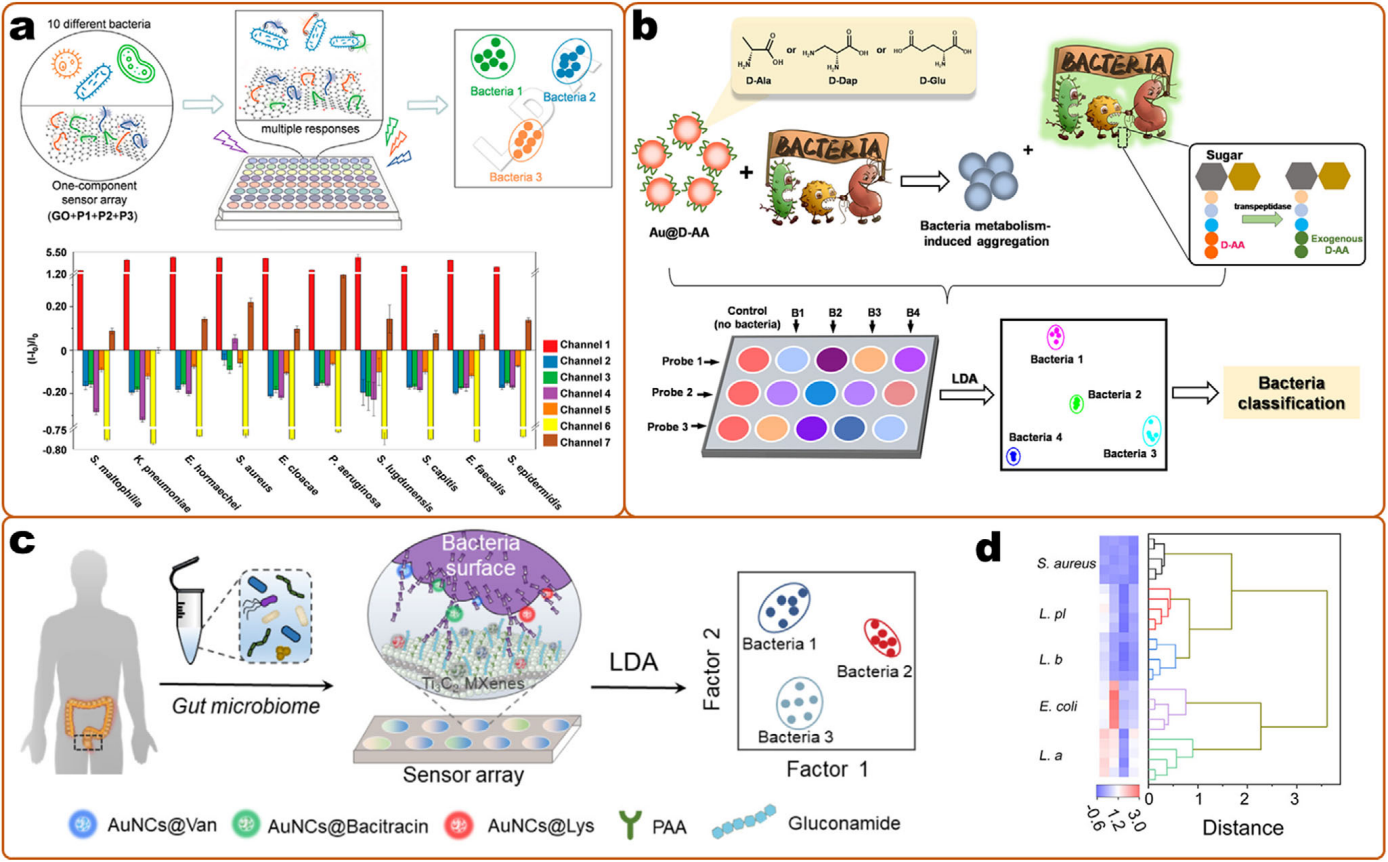
Illustrative Examples of Multichannel Sensor Arrays for Bacteria Detection. a) Multichannel Sensor Array for Rapid Bacteria Identification: Utilizes a modified PEI‐GO complex, where nonspecific discrimination is based on electrostatic and hydrophobic interactions. The sensor array generates unique fingerprint patterns for each bacterium through multichannel fluorescence responses. Reproduced with permission.^[^ ^597^ ^]^ Copyright 2022, American Chemical Society. b) Metabolism‐Triggered Colorimetric Sensor Array for Bacteria Fingerprinting: Based on the differential metabolic capabilities of bacteria toward various D‐AAs, which result in distinct aggregation states (colors) of gold nanoparticles. Reproduced with permission.^[^ ^600^ ^]^ Copyright 2022, American Chemical Society. (c, d) Multichannel Sensor Array for Gut Microbiota Sensing: c) The array leverages the recognition ability of different antimicrobial agents and the competitive binding between gold nanoclusters and bacteria toward MXenes. d) The resulting heat map and HCA dendrogram showcase the fluorescent fingerprint patterns of five bacterial strains. Reproduced with permission.^[^ ^601^ ^]^ Copyright 2023, American Chemical Society.

Wang et al. reported a sensor array composed of electrostatic complexes, including negatively charged graphene oxide (GO) and polyethyleneimine (PEI)‐based fluorescent probes, which successfully demonstrated a distinctive multimode response to 10 bacteria.^[^ ^597^ ^]^ The fluorescence of the probes was initially quenched by GO, but the interactions based on electrostatic forces and other nonspecific bindings—such as hydrophilic, hydrophobic, polar, and aromatic functional groups between bacteria and probes—recovered the fluorescence. This sensor array generated signal patterns with seven outputs, allowing for the identification of bacteria with 100% accuracy (**Figure** **14**a). However, nonspecific interactions can be easily blocked by surrounding factors, especially in real‐world samples, leading to limited identification capability and sensitivity. The metabolism of D‐amino acids (D‐AAs) is a specific reaction in bacteria. Exogenous D‐AAs can be specifically incorporated into the peptidoglycan in bacterial cell walls by bacterial D,D‐transpeptidases, a process that does not occur in fungi, mammalian cells, or other substances.^[^ ^598^, ^599^ ^]^

Gao et al. developed a colorimetric sensor array utilizing three types of D‐AA‐modified gold nanoparticles—D‐glutamate, D‐alanine, and D‐2,3‐diaminopropionic acid—as recognition units (Figure 14b) for detecting multiple bacteria. In the presence of bacteria, the D‐AAs on the surface of the gold nanoparticles are displaced, leading to the instability and aggregation of the nanoparticles. Due to the disparity in bacterial metabolic abilities, diverse aggregation behaviors are produced, creating unique responses for each bacterium.^[^ ^600^ ^]^ The receptors in the recognition units are crucial for balancing sensitivity and multiplex analysis capability. Higher affinity and stronger interaction can enhance multiplex sensing ability, but this can also reduce sensitivity. Therefore, key challenges include increasing the robustness and effectiveness of receptors in complex environments. Antimicrobial agents and competitive reactions can enhance anti‐interference capabilities and help achieve this balance. Antimicrobial agents bind to different donors on the bacterial cell membrane surface, while competitive reactions limit the participation of certain molecules in the reaction. Combining these two methods can significantly improve the performance of the sensor array. Liu et al. reported a robust and rapid multichannel sensor array utilizing three antimicrobial agents (lysozyme, vancomycin, and bacitracin) modified with three gold nanoclusters (AuNCs) having different fluorescent emissions, along with gluconamide‐functionalized Ti₃C₂ MXenes, to produce superior fingerprint patterns of bacteria (Figure 14c).^[^ ^601^ ^]^ The lysozyme, vancomycin, and bacitracin specifically bind to polysaccharides, D‐Ala‐D‐Ala moieties, and pyrophosphate groups on the bacterial cell surface,^[^ ^602^ ^]^ respectively. This setup allows bacteria to be competitively absorbed by the AuNCs toward the MXenes, altering the interaction between MXenes and AuNCs. This results in the recovery or further inhibition of the fluorescence signals of AuNCs.

The fluorescence responses vary significantly depending on the characteristics of the bacteria and their interactions with the different antibiotics. This variation allows for the creation of distinct fluorescence patterns, enabling accurate identification and differentiation of bacterial species.

**Figure 15:**
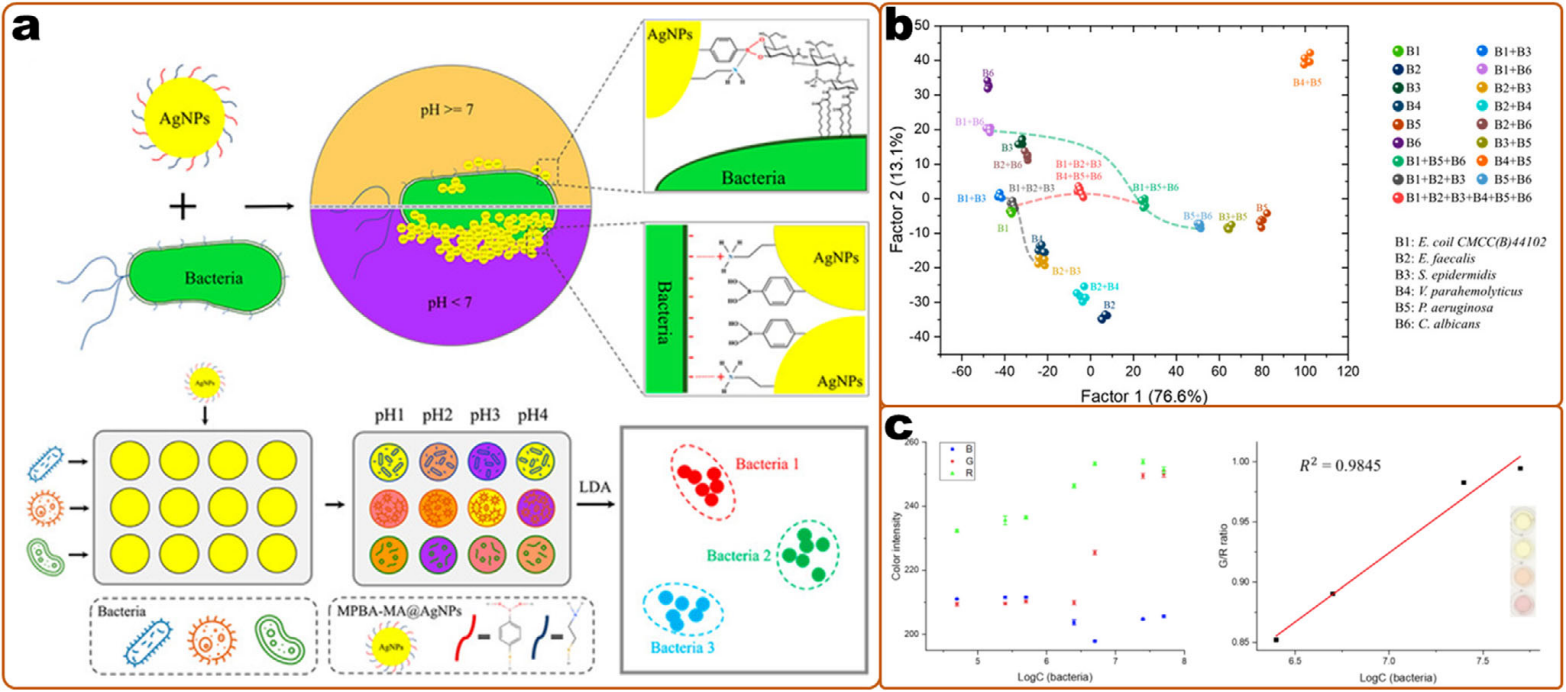
Colorimetric Sensor Array for Bacteria Discrimination. a) Principles of Colorimetric Sensor Array: Based on Wulff‐type boronate and electrically charged functionalized AgNPs at different pH levels for bacterial discrimination. b) Canonical Score LDA Plot for Bacterial Mixtures: Each point represents the fingerprint pattern of a bacterial sample, illustrating the separation and discrimination of different bacterial mixtures. c) RGB Values and Concentration Relationship: The RGB values of functionalized AgNPs reacting with *P. aeruginosa* at different concentrations, showing the linear relationship between the RGB values and the concentration of *P. aeruginosa*. Reproduced with permission.^[^ ^603^ ^]^ Copyright 2019, American Chemical Society.

The sensor array realizes the multiplex detection of a variety of bacteria, providing significant opportunities for sensor development. From the previous examples, we see that current sensor arrays can only discriminate bacteria in an existing database. Few studies have explored unknown microorganisms, and interfering species may also cause significant deviations in results. Deep research is still needed to promote real‐world applications. Yan et al. explored the possibility of discriminating complex bacterial mixtures and achieving quantitative detection with a colorimetric sensor array (**Figure** **15**).^[^ ^603^ ^]^ They used silver nanoparticles (AgNPs) modified with Wulff‐type 4‐mercaptophenylboronic acid (MPBA) and mercaptoethylamine (MA), which had varying affinities to bacteria based on specific interactions (boronic acid to cis‐diol) and electrostatic interactions at different pH levels. The AgNPs exhibited different colors in various detection channels. To some extent, the sensor array could distinguish single bacteria, pairwise mixtures, and multiple mixtures. They also used the sensor array to detect P. aeruginosa at different concentrations, achieving semi‐quantitative detection based on RGB values.

**Figure 16:**
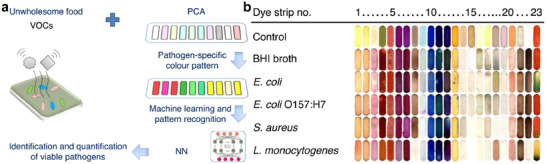
Detection of Various Pathogens Based on Volatile Organic Compounds. a) Principle of Paper Chromogenic Array: Illustration of the paper chromogenic array for multiplex pathogen detection, utilizing machine learning and automatic pattern recognition. b) Signal Patterns from Various Pathogens: Representation of the distinct signal patterns generated by different pathogens, enabling their identification and discrimination. Reproduced with permission.^[^ ^117^ ^]^ Copyright 2021, Springer Nature.

Many sensor arrays rely on the recognition of surface properties of bacteria. However, pathogen identification can also be achieved by detecting volatile organic compounds (VOCs) emitted by the pathogens, which concentrate on signature metabolic biomarkers.^[^ ^604^, ^605^ ^]^ Colorimetric arrays targeting VOCs have enabled the detection of unwanted bacteria in processed meat and seafood.^[^ ^606^, ^607^, ^608^ ^]^ However, challenges remain in single‐pathogen detection due to limitations in dye selection, data analysis, and various background signals. Addressing these issues, Yang and collaborators reported a novel nondestructive method for pathogen detection using a paper chromogenic array enhanced by ML.^[^ ^117^ ^]^ The paper chromogenic array consisted of 23 paper strips soaked in chromogenic dyes and dye combinations, which form combinational color changes upon contact with VOCs emitted by pathogens (**Figure** **16**). Due to the large volume of data generated—various signal units with different pathogens and at different infection times—visual inspection and decoding are complicated and time‐consuming. To address this, the authors utilized a trained multi‐layer neural network in conjunction with the 23 dye strips. This system achieved high accuracy in pathogen identification (95%) and quantification (93%). This approach offers several advantages: it is a nondestructive method for detecting pathogens in food, requires no sample preparation steps such as enrichment, culturing, or incubation, and holds great promise for food safety applications.

From a practical perspective, nanosensors should have fewer components and operation steps to simplify their use. For instance, eliminating the need for recognition elements can be advantageous. Raman spectroscopy, known for its remarkable fingerprinting capabilities, can perform label‐free identification. A single‐cell Raman spectrum (SCRS) represents the biochemical fingerprints of individual cells, capturing the characteristic vibrational frequencies of lipids, proteins, and nucleic acids. This provides a snapshot of bacterial phenotypes, also referred to as a “Raman phenotype”.^[^ ^165^, ^609^ ^]^ Raman spectroscopy offers significant advantages over traditional biochemical strategies, as it enables phenotype identification without the need for specifically designed labels and probes. Given the diversity of bacterial species and phenotypes within a sample, achieving single‐cell resolution necessitates the use of confocal Raman spectroscopy. Some researchers have successfully employed Raman spectroscopy to identify bacteria.^[^ ^165^, ^610^ ^]^ However, several core challenges need to be addressed with this strategy. Different bacterial phenotypes often have similar molecular structures and compositions, resulting in subtle variations in their corresponding Raman spectra. Accurately distinguishing these subtle spectral differences is critical for the effective identification and characterization of bacterial phenotypes.

Spontaneous Raman scattering efficiency is inherently weak (≈10⁻⁷–10⁻⁸), necessitating high signal‐to‐noise ratios (SNRs) to achieve accurate identification, which in turn requires long measurement times (≈15–60 seconds per spectrum). Additionally, the vast diversity of pathogenic bacteria, encompassing numerous species and strains, demands the acquisition of extensive spectral datasets. ML has become integral to this method. Ho and coworkers trained a CNN using a large dataset of bacterial Raman spectra, enabling the differentiation of 30 common bacterial pathogens.^[^ ^611^ ^]^ To address the issues of long signal acquisition times and low SNRs, Huang's group developed a series of standard Raman operation protocols aimed at achieving optimal and accurate identification of bacterial phenotypes.^[^ ^612^ ^]^ Using SNR as a standard criterion with short acquisition times, Huang's group produced 11,141 SCRS and applied two ML methods—t‐SNE and LDA—to analyze the datasets. This approach enabled ultra‐fast and precise bacterial classification.

Compared with Raman spectra, SERS provides higher sensitivity, allowing for the suppression of fluorescence background and reducing the influence of bacterial physiological state, growth stage, or culture conditions.^[^ ^613^ ^]^ Shang et al. investigated the influence of Ag nanoparticle binding time and concentration on the differentiation of eight beer spoilage bacteria.^[^ ^614^ ^]^ Later, the SERS spectra were collected under optimal conditions. The t‐SNE method was used for downscaling analysis, and machine learning algorithms, including SVM, KNN, and LDA, were employed for analysis and prediction, achieving accuracy rates above 90%. Additionally, Li's group reported the accurate identification of six pathogenic Vibrio species in contaminated seafood using neural network‐assisted SERS.^[^ ^615^ ^]^ They used a portable Raman spectrometer and Au@Ag nanoparticles as the enhanced substrate, which provided superior Raman enhancement compared to other metal nanoparticles. By comparing seven traditional ML methods, they found that CNN delivered the best performance with an accuracy of 99.7%, and the entire detection process could be completed in 15 minutes. In this strategy, ML algorithms are essential for discriminating pathogens during data processing. However, challenges remain in achieving efficient and uniform SERS enhancement, particularly in liquid Raman measurements.^[^ ^616^ ^]^

A summary of multi‐microorganism analyses is provided in **Table** **6**.

**Table 6:** Summary of nanosensors for detecting multiple bacteria.

Analyte	Sensor system	Detection limit	Environment	Refs.
*E. coli O157:H7, S. paratyphi B, S. enteritis, S. paratyphi C, S. choleraesuis*	An optical sensor with multicolor up‐conversion nanoparticles	10^5^ cfu/mL (*E. coli O157:H7*), 10^5^ cfu/mL (*S. paratyphi B*), 10^7^ cfu/mL (*S. enteritis*), 10^5^ cfu/mL (*S. paratyphi C*), 10^6^ cfu/mL (*S. choleraesuis*)	Buffer solutions	[568]
*E. coli O157:H7, Salmonella choleraesuis, L. monocytogenes*	A multicolor and ultrasensitive enzyme‐linked immunosorbent assay platform based on the fluorescence hybridization chain reaction and gold	3.4 × 10^1^ cfu/mL (*E. coli O157:H7*), 6.4 × 10° cfu/mL (*Salmonella choleraesuis*), 7.0 × 10^1^ cfu/mL (*L. monocytogenes*)	Milk	[572]
H9N2, H1N1, H7N9 avian influenza virus	An optical sensor based on fluorescent magnetic multifunctional nanospheres	0.02 pg/mL	Buffer solutions, serum	[575]
Influenza A H1N1 virus human adenovirus (HAdV)	A lateral flow immunoassay strip based on Fe_3_O_4_@Ag nanoparticles	50 pfu/mL (H1N1), 10 pfu/mL (HAdV)	Buffer solutions	[576]
Virus	A combinatorial arrayed reactions for multiplexed evaluation of nucleic acids	Qualitative Identification	Infected samples	[590]
*Staphylococcus aureus (CD‐35), Escherichia coli (CD‐2), Bacillus subtilis (FD6b), Pseudomonas aeruginosa (ATCC 19660)*,	A sensor array based on fluorescent polymers	Qualitative Identification	Buffer solutions	[594]
10 bacteria	A sensor array based on polyethyleneimine and graphene oxide	Working range, 0.025 to 1 (OD_600_)	Water, urine	[597]
*Staphylococcus aureus*, *Escherichia coli*, *E. coli DH5α*, *Salmonella typhimurium*, *Listeria monocytogenes*, *Cronobacter sakazakii*, methicillin‐resistant *Staphylococcus aureus*, kanamycin‐resistant *Escherichia coli*	A sensor array based on D‐amino acid‐modified gold nanoparticles	Qualitative Identification	Buffer solutions	[600]
*Lactobacillus acidophilus, Lactobacillus brevis, Lactobacillus plantarum, Escherichia coli, Staphylococcus aureus*	A sensor with antimicrobial agent (vancomycin, bacitracin, and lysozyme) functional gold nanoclusters and gluconamide‐modified Ti_3_C_2_ MXenes	Working range, 0.005 to 0.05 (OD_600_)	Buffer solutions	[601]

## 5 Conclusion and Future Challenges

In recent years, nano‐sensing has experienced rapid advancements in both analytical techniques and sensing materials. Multiplex detection has demonstrated exceptional performance in addressing a broad range of analytical challenges. By employing diverse chemo‐responsive molecules, biological receptors, or label‐free methods in conjunction with chemometric analysis and ML algorithms, traditional “one‐to‐one recognition” models—where a highly specific receptor is required for each analyte—have become less efficient and fall short of the capabilities offered by artificial taste and olfaction systems. These conventional methods struggle to accommodate the vast number of analytes or complex mixtures that require detection, limiting their ability to provide comprehensive analysis.

The emergence of sensors with multiplex detection capabilities offers alternative approaches, including the integration of multiple recognition elements, sensor arrays, multiple sensing channels, and label‐free detection enhanced by ML algorithms. Recent advances in nano‐sensing have made it possible to achieve sensitive and reliable detection of single or multiple analytes across a variety of sample types—whether gaseous, aqueous, chemical, or biological. A key advantage of multiplex detection is its ability to generate multiple channel responses, offering composite data that can discriminate between highly similar analytes, even in complex mixtures.

Beyond the integration of recognition elements and the use of multiple channels, sensor arrays, and label‐free strategies focus on detecting the inherent chemical properties of analytes. These approaches provide highly specific responses to a wide range of analytes, often resulting in high‐dimensional data. The application of ML to analyze such data opens up new possibilities for applying nano‐sensing technologies to practical and real‐world samples. However, challenges remain in further enhancing the sensitivity, selectivity, and scalability of these systems to fully realize their potential in complex environments. Continued innovation in this field will be essential to overcome these challenges and expand the practical applications of nano‐sensing technologies. Based on multiplex detection, numerous analytes of concern—including ions, chemicals, and microorganisms—can be detected even at much lower concentrations, effectively reducing false positives often associated with traditional “one‐to‐one” detection methods. Achieving ideal performance in nanosensing is a systematic endeavor, involving multiple facets beyond just recognition strategies and data analysis. This article primarily focuses on those aspects, but it is important to acknowledge other critical factors that contribute to the efficacy of nanosensors, such as the orientation of recognition elements,^[^ ^617^ ^]^ the stability of nanocomposites,^[^ ^618^ ^]^ the signal response,^[^ ^619^ ^]^ and signal enhancement.

While multiplex detection has made significant strides, it generally provides a one‐by‐one analysis for composite samples, and it has yet to achieve the capability of complete quantitative analysis for all components within a sample. In practice, the interest often lies not in identifying every single component, but rather in obtaining information about flavor, place of origin, quality, and potential contaminants. Multiplex detection offers substantial possibilities for realizing these practical goals, bringing us closer to more comprehensive and meaningful analyses.

Increasing the performance of multiplex detection in practical applications remains the most significant challenge. While substantial success has been achieved in controlled laboratory environments, widespread field use is still a distant goal. To confirm the identity of unknown analytes effectively, improvements in reproducibility, prediction accuracy, and the elimination of interferents are essential. Paper‐based test strips, microfluidic devices, and other POCT techniques have made progress in this direction, beginning to adapt to the demands of real‐world applications.

Looking ahead, the continued development of nanosensing technologies will require addressing these challenges and refining techniques to enhance their accuracy, sensitivity, and applicability in complex environments. As the field evolves, the integration of advanced recognition strategies, robust data analysis, and enhanced signal processing will be critical to unlocking the full potential of multiplex detection in practical, real‐world settings. By overcoming these obstacles, nanosensing can achieve broader adoption and become a reliable tool for diverse applications, from food safety to environmental monitoring and beyond.

The future of food analysis is poised to evolve significantly with the integration of nanosensor technologies, machine learning, and other advanced analytical tools. While the application of machine learning has shown great promise in enhancing the data analysis and predictive capabilities of nanosensors, it is essential to envision a more comprehensive framework that integrates these technologies with other innovative approaches. Such as integrating multi‐omics integration and data‐driven risk prediction models which can identify unknown contaminants, strengthen early‐warning capabilities, and help prevent incidents like the “melamine incident”. The next generation of food analysis will likely involve a synergy between high‐throughput nanosensor platforms, real‐time monitoring systems, and decentralized data analytics, creating a network of intelligent food safety management tools.

Another important direction for future research is the integration of nanosensors with Internet of Things (IoT) platforms and blockchain technology. This could enable the real‐time tracking of food quality throughout the entire supply chain, from farm to fork, ensuring transparency and accountability at each stage. Such an integrated approach could significantly reduce the risk of food fraud and contamination, providing more comprehensive protection for consumers.

Furthermore, as the demand for sustainable and safe food increases globally, there will be a growing need for sensors that not only detect contaminants but also provide insights into food quality, freshness, and nutritional content. Multi‐functional sensors capable of assessing these parameters simultaneously would be highly beneficial in supporting the food industry's move toward more transparent and consumer‐centric practices.

Overall, the future of food analysis will be shaped by an interdisciplinary approach that integrates advanced sensor technologies, AI and machine learning, real‐time data analytics, and sustainable practices. This convergence will not only enhance our ability to detect and prevent food safety issues but also revolutionize the entire food supply chain, leading to a safer, more sustainable, and more transparent global food system.

## Conflict of Interest

The authors declare no conflict of interest.
